# The Lipid Metabolism as Target and Modulator of BOLD‐100 Anticancer Activity: Crosstalk with Histone Acetylation

**DOI:** 10.1002/advs.202301939

**Published:** 2023-09-26

**Authors:** Dina Baier, Theresa Mendrina, Beatrix Schoenhacker‐Alte, Christine Pirker, Thomas Mohr, Mate Rusz, Benedict Regner, Martin Schaier, Nicolas Sgarioto, Noël J.‐M. Raynal, Karin Nowikovsky, Wolfgang M. Schmidt, Petra Heffeter, Samuel M. Meier‐Menches, Gunda Koellensperger, Bernhard K. Keppler, Walter Berger

**Affiliations:** ^1^ Center for Cancer Research and Comprehensive Cancer Center Medical University Vienna Borschkegasse 8a Vienna 1090 Austria; ^2^ Institute of Inorganic Chemistry University of Vienna Waehringer Str. 42 Vienna 1090 Austria; ^3^ Research Cluster “Translational Cancer Therapy Research” Vienna 1090 Austria; ^4^ Joint Metabolome Facility University of Vienna and Medical University of Vienna Waehringer Str. 38 Vienna 1090 Austria; ^5^ ScienceConsult Guntramsdorf 2351 Austria; ^6^ Institute of Analytical Chemistry Faculty of Chemistry University of Vienna Waehringer Str. 38 Vienna 1090 Austria; ^7^ Anna Spiegel Center of Translational Research Department of Medicine I Medical University Vienna Lazarettgasse 14 Vienna 1090 Austria; ^8^ Départment de pharmacologie et physiologie Faculté de médecine Centre de recherché de l hôpital Université de Montréal Saint‐Justine (7.17.020), 3175 Chemin de la Côte Ste‐Catherine Quebec H3T1C5 Canada; ^9^ Unit of Physiology and Biophysics Department of Biomedical Sciences University of Veterinary Medicine Vienna Veterinaerplatz 1 Vienna 1210 Austria; ^10^ Neuromuscular Research Department Center for Anatomy and Cell Biology Medical University of Vienna Währinger Str. 13 Vienna 1090 Austria

**Keywords:** BOLD‐100, chemoresistance, histone acetylation, lactate transporter, lipid metabolism, mitochondrial respiration, ruthenium

## Abstract

The leading first‐in‐class ruthenium‐complex BOLD‐100 currently undergoes clinical phase‐II anticancer evaluation. Recently, BOLD‐100 is identified as anti‐Warburg compound. The present study shows that also deregulated lipid metabolism parameters characterize acquired BOLD‐100‐resistant colon and pancreatic carcinoma cells. Acute BOLD‐100 treatment reduces lipid droplet contents of BOLD‐100‐sensitive but not ‐resistant cells. Despite enhanced glycolysis fueling lipid accumulation, BOLD‐100‐resistant cells reveal diminished lactate secretion based on monocarboxylate transporter 1 (MCT1) loss mediated by a frame‐shift mutation in the MCT1 chaperone basigin. Glycolysis and lipid catabolism converge in the production of protein/histone acetylation substrate acetyl‐coenzymeA (CoA). Mass spectrometric and nuclear magnetic resonance analyses uncover spontaneous cell‐free BOLD‐100‐CoA adduct formation suggesting acetyl‐CoA depletion as mechanism bridging BOLD‐100‐induced lipid metabolism alterations and histone acetylation‐mediated gene expression deregulation. Indeed, BOLD‐100 treatment decreases histone acetylation selectively in sensitive cells. Pharmacological targeting confirms histone de‐acetylation as central mode‐of‐action of BOLD‐100 and metabolic programs stabilizing histone acetylation as relevant Achilles’ heel of acquired BOLD‐100‐resistant cell and xenograft models. Accordingly, histone gene expression changes also predict intrinsic BOLD‐100 responsiveness. Summarizing, BOLD‐100 is identified as epigenetically active substance acting via targeting several onco‐metabolic pathways. Identification of the lipid metabolism as driver of acquired BOLD‐100 resistance opens novel strategies to tackle therapy failure.

## Graphical abstract

In this study, the clinically investigated first‐in‐class anti‐Warburg ruthenium complex BOLD‐100 is identified as epigenetic gene expression modulator based on a complex interference with the cancer metabolome. BOLD‐100 interferes with glycolysis reducing cellular lactate secretion, lipid stores, and histone acetylation. Deregulation of lipid metabolism is identified as major driver of acquired BOLD‐100 resistance, opening new strategies to overcome therapy failure.

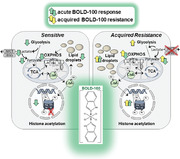

## 1 Introduction

The ruthenium anticancer complex BOLD‐100 (sodium trans‐[tetrachlorobis(1H‐indazole)ruthenate(III)] with cesium as an intermediate salt form; predecessor molecules: IT‐139/NKP‐1339/KP1339) has shown promising in vitro and in vivo efficacy against various types of solid tumors.^[^ ^1^ ^]^ In clinical phase I evaluation, BOLD‐100 therapy led to disease stabilization or even partial response in several types of advanced solid tumors including colorectal cancer (CRC), non‐small cell lung cancer, and neuroendocrine tumors of carcinoid origin.^[^ ^2^ ^]^ In addition, an overall excellent tolerability was observed. Recently, BOLD‐100 successfully completed a phase Ib clinical trial in combination with folinic acid, fluorouracil, and oxaliplatin (FOLFOX regimen) (*NCT04421820*) and is currently undergoing global clinical phase II evaluation for the treatment of advanced gastrointestinal cancers (CRC, pancreatic, gastric, and cholangiocarcinoma). Yet, a general limitation of systemic cancer therapy efficacy presents the acquisition of treatment resistance.^[^ ^3^ ^]^ Hence, the dissection of underlying resistance mechanisms during (pre)clinical assessment of novel anticancer drugs is inevitable. Accumulating evidence suggests that acquired therapy resistance of diverse cancer types depends on adaptations of metabolic processes including, besides shifts toward a glycolytic phenotype^[^ ^4^ ^]^ or enhanced glutaminolysis,^[^ ^5^ ^]^ changes of lipid *de novo* synthesis, turnover, and uptake mechanisms.^[^ ^6^ ^]^ Understanding these complex and intertwined mechanisms of cell metabolic reprogramming may uncover vulnerabilities exploitable by specific targeting. Mechanistically, BOLD‐100 is an albumin‐binding complex^[^ ^7^ ^]^ that hitchhikes this major serum protein leading to selective accumulation in the tumor via the enhanced permeability and retention (EPR) effect.^[^ ^8^ ^]^ Inside the tumor, Ru(III) is proposed to be activated by reduction to Ru(II) due to prevalent reductive conditions. BOLD‐100 is a versatile small molecule with manifold intracellular modes‐of‐action summarized previously by our group.^[^ ^9^ ^]^ Additionally, BOLD‐100 was recently identified to target ribosomal proteins.^[^ ^10^ ^]^ Most prominent, BOLD‐100 is a potent endoplasmic reticulum (ER) stress inducer via the disruption of the glucose‐regulated protein 78 (GRP78, BIP) response,^[^ ^1^, ^8^ ^]^ the master chaperone in the ER and negative regulator of the unfolded protein response (UPR).^[^ ^11^ ^]^

An estimation about the cellular lipid load can be obtained from the cytosolic lipid droplet (LD, adiposome) content.^[^ ^12^ ^]^ LDs consist of an outer phospholipid layer enclosing neutral lipids, mainly triacylglycerides (TAG) and cholesterol esters.^[^ ^13^ ^]^ Hence, LDs are the major cellular fat storage compartments providing an important reservoir of metabolic building blocks. Fatty acids can be mobilized from LDs upon demand for energy production via ß‐oxidation or anabolic processes such as membrane biosynthesis.^[^ ^14^ ^]^ A dysregulation of the intracellular lipid homeostasis as a consequence of prolonged ER stress/UPR has been reported to cause alternative transcription of lipid metabolism‐associated genes,^[^ ^15^ ^]^ a disturbed lipid *de novo* synthesis,^[^ ^16^ ^]^ as well as altered intracellular lipid accumulation.^[^ ^17^ ^]^

In recent years, several studies revealed that the lipid metabolism substantially affects chemosensitivity^[^ ^18^ ^]^ and that the metabolic state could even predict for (chemo)therapy response or development of resistance.^[^ ^19^ ^]^ Regarding acquired resistance especially against non‐platinum metal‐based chemotherapy, the specific molecular mechanisms underlying cellular metabolic changes remain widely enigmatic. Lately, we uncovered distinct differences between acquired platinum and ruthenium resistance in terms of metabolic reprogramming. In detail, cancer‐specific genome‐scale metabolic modelling detected changes in the amino acid (AA) as well as fatty acid metabolism as distinctly varying determinant of the metabolic phenotype closer associated with acquired BOLD‐100 as compared to oxaliplatin resistance.^[^ ^20^ ^]^

In a recent publication, we identified BOLD‐100 as anti‐Warburg drug, depleting HCT116 cells of lactate and pyruvate by interference with glycolysis.^[^ ^21^ ^]^ Accordingly, enhanced glycolytic activity was associated with resistance development against the ruthenium complex. An underestimated aspect of metabolic perturbation and adaptation in response to (metal‐based chemo)therapy or in acquired resistance is the close connection with epigenetic gene expression regulation. Acetylated coenzyme A (CoA) is a key metabolite produced from several metabolic sources that participates as acetyl group donor in histone acetylation executed by histone acetyltransferases (HAT).^[^ ^22^ ^]^ Acetyl‐CoA‐generating metabolites include monocarboxylates such as lactate or pyruvate, taken up by monocarboxylate transporters (MCT) or produced via glycolysis.^[^ ^23^ ^]^ Expression and activity of MCTs require the presence of a chaperone, transmembrane glycoprotein CD147 (basigin, *BSG*).^[^ ^24^ ^]^ Interdependently, CD147 expression relies on MCT expression.^[^ ^25^ ^]^ Other fuels of acetyl‐CoA comprise acetate or fatty acids.^[^ ^26^ ^]^ Hence, acetyl‐CoA connects the tricarboxylic acid (TCA) cycle with lipid synthesis or histone acetylation as well as lipid catabolism via ß‐oxidation and the TCA cycle.^[^ ^27^ ^]^ This study provides an in‐depth analysis of BOLD‐100‐induced metabolic perturbations and their role in acquired resistance development against the ruthenium complex.

## 2 Results

### 2.1 Acquired BOLD‐100 Resistance is Associated with an Altered Lipid Metabolism Gene Expression Pattern

**Figure 1:**
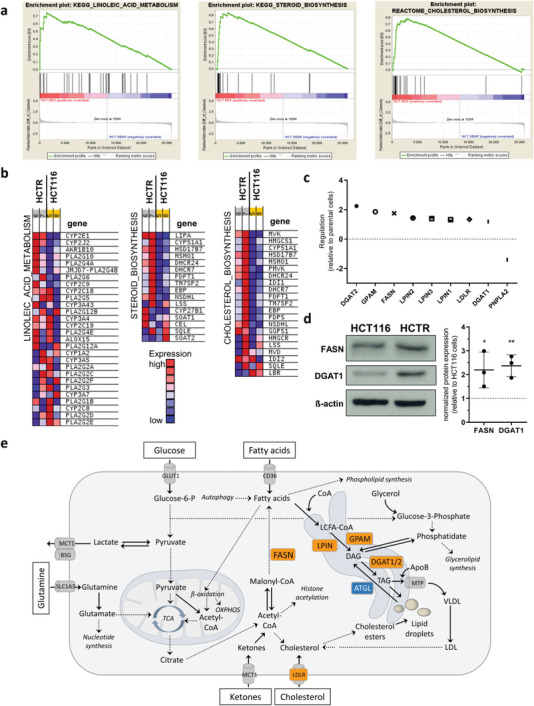
Acquired BOLD‐100 resistance of HCT116 cells is associated with a deregulation of the lipid metabolism. a) GSEA identifies “LINOLEIC_ACID_METABOLISM” (nominal *p*‐value < E^−7^; FDR *q*‐value = 0.022) and “STEROID_BIOSYNTHESIS” (nominal *p*‐value < E^−7^; FDR *q*‐value = 0.011) as the first and second top enriched gene sets, respectively, from the KEGG database in HCTR versus HCT116 cells. In the Reactome database, “CHOLESTEROL_BIOSYNTHESIS” (nominal *p*‐value = 0.002; FDR *q*‐value = 0.027) was identified as top fourth upregulated gene set. b) Heatmaps of respective top enriched gene sets identified from GSEA in HCTR versus HCT116 cells. c) Regulation of specific genes involved in fatty acid and cholesterol metabolism of HCTR versus HCT116 cells (indicated as dashed line at 0). mRNA levels with a fold change >│1.1│ were considered for graphical display. d) FASN and DGAT1 proteins in whole cell lysates of HCTR versus parental HCT116 cells analyzed by Western blotting. β‐actin served as loading control. Unpaired two‐tailed student´s t‐test: *p<0.05, **p<0.05. e) Scheme displaying the cellular pathways leading to lipid droplet formation. Genes/proteins upregulated in HCTR versus HCT116 cells are depicted in orange while downregulated ATGL (PNPLA2) is given in blue. Building blocks for schematic representation were provided by Motifolio (license holder: Thomas Mohr).

Acquired BOLD‐100‐resistant CRC HCT116 (HCTR) and pancreatic cancer Capan‐1 (CapanR) cells were previously established from their respective sensitive parental counterparts.^[^ ^21^ ^]^ On the transcriptional level, changes of mRNA expression profiles of HCTR or CapanR compared to their respective parental cells were determined by whole genome gene expression analyses followed by Gene Set Enrichment Analyses (GSEA). Of the top 20 enriched gene sets in HCTR cells identified in the KEGG database, five gene sets were associated with the lipid metabolism, with “LINOLEIC_ACID_METABOLISM” (nominal *p*‐value < E^−7^; false discovery rate (FDR) *q*‐value = 0.022) and “STEROID_BIOSYNTHESIS” (nominal *p*‐value < E^−7^; FDR *q*‐value = FDR 0.011) as the first and second top‐enriched gene sets, respectively (**Figure** **1**a). Additionally, in the Reactome database “CHOLESTEROL_BIOSYNTHESIS” (nominal *p*‐value = 0.002; FDR *q*‐value = 0.027) was identified as top fourth upregulated gene set. The corresponding heatmaps revealed upregulation of defined mRNAs coding for members of the cytochrome P450 (*CYP*) oxidase system, involved in drug metabolism and lipid synthesis,^[^ ^28^ ^]^ lysosomal lipase A (*LIPA*), and the sterol synthesis key enzyme mevalonate kinase (*MVK*)^[^ ^29^ ^]^ (Figure 1b). In CapanR cells, the lipid metabolism‐related gene sets “OXIDATIVE_PHOSPHORYLATION” (nominal *p*‐value < E^−7^; FDR *q*‐value = 0.121), “TERPENOID_BACKBONE_BIOSYNTHESIS” (nominal *p*‐value = 0.169; FDR *q*‐value = 0.652), and “STEROID_BIOSYNTHESIS” (nominal *p*‐value = 0.4257; FDR *q*‐value = 0.784) appeared as third, sixth, and twentieth top enriched, respectively, in the KEGG database (Figure S1a, Supporting Information). Corresponding heatmaps showed upregulated expression of genes encoding for *LIP* *A* as well as cytochrome c oxidase family members involved in oxidative phosphorylation (Figure S1b, Supporting Information).^[^ ^30^ ^]^ In‐depth screening of lipid metabolism‐associated genes in HCTR versus HCT116 cells identified a higher expression of specific *de novo* lipid synthesis and lipid droplet biogenesis genes, namely diglyceride acyltransferase (*DGAT1/2*), glycerol‐3‐phosphate acyltransferase (*GPAT/GPAM*), fatty acid synthase (*FASN*), the family of lipins (*LPIN1/2/3*), and the low‐density lipoprotein receptor (*LDLR*) in the acquired resistant subline (Figure 1c). Contrary, expression of the gene encoding for patatin‐like phospholipase domain‐containing 2 (*PNPLA2*, *ATGL*) involved in TAG catabolism^[^ ^14^ ^]^ was downregulated in BOLD‐100‐resistant cells, supporting a lipid anabolic rather than catabolic resistance‐associated phenotype in the CRC model. CapanR cells were characterized by enhanced mRNA expression of microsomal triglyceride transfer protein (*MTTP*), the fatty acid importer *CD36*, and *PNPLA2* together with a decrease of *FASN*, *GPAT2*, and *DGAT2* (Figure S1c, Supporting Information), indicating a switch toward enhanced lipid uptake, intracellular trafficking and utilization upon BOLD‐100 resistance development in the pancreatic cancer model. In line with transcriptional regulation, protein expression of FASN and DGAT1 was significantly upregulated in HCTR compared to parental cells (Figure 1d). A scheme providing an overview of the pathways leading to lipid storage compartment biogenesis indicating transcriptional and translational alterations associated with acquired BOLD‐100 resistance is provided in Figure 1e.

### 2.2 Impact of BOLD‐100 Exposure and Acquired Resistance on Cellular Lipid Storage Compartments

**Figure 2:**
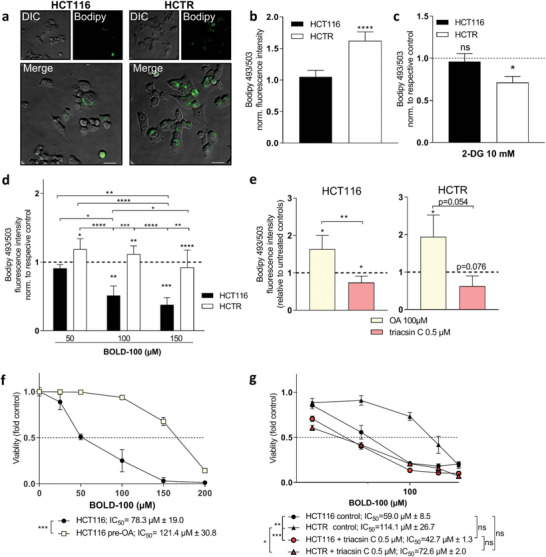
Crosstalk between BOLD‐100 exposure, acquired resistance and cellular lipid storage compartments. a) Representative live cell images of HCT116 and HCTR cells stained for LDs with 0.5 µM Bodipy 493/503 (green fluorescence). Small upper left images: bright field, right images: Bodipy 493/503 staining, big images: merged pictures, scale bar: 50 µm b) Normalized fluorescence intensity of HCT116 and HCTR cells determined by flow cytometry analyses (FACS) after staining with 0.5 µM of Bodipy 493/503. Unpaired two‐tailed student´s t‐test: ****p<0.0001. c) Fluorescence intensity of HCT116 and HCTR cells treated with 2‐DG for 72 h determined by FACS after staining with 0.5 µM of Bodipy 493/503 for 15 min. Data are normalized to the respective controls. Two‐way ANOVA with Tukey´s multiple comparisons test: *p<0.05. d) Fluorescence intensity of HCT116 and HCTR cells treated with the indicated concentrations of BOLD‐100 for 72 h determined by FACS after staining with 0.5 µM of Bodipy 493/503 for 15 min. Data are normalized to the respective controls. Two‐way ANOVA with Tukey´s multiple comparisons test: *p<0.05, **p<0.005, ***p<0.0005, ****p<0.0001. e) Bodipy 493/503 fluorescence intensity of parental HCT116 or HCTR cells upon 72 h treatment with 100 µM of oleic acid (OA) for LD induction or 0.5 µM of triacsin C for LD reduction. One‐way ANOVA with Tukey´s multiple comparisons test: *p<0.05, **p<0.005. f) Endogenous LD levels were increased by pre‐treatment with 100 µM of OA for 72 h. Cell viability was determined by 3‐(4,5‐dimethylthiazol‐2‐yl)−2,5‐diphenyltetrazolium bromide (MTT) assay after 72 h of exposure to BOLD‐100 at indicated concentrations. Two‐way ANOVA with Bonferroni's multiple comparison test: ***p<0.001. g) Cells were pre‐treated with 0.5 µM triacsin C for 24 h for the reduction of LD levels and BOLD‐100 was spiked in at indicated concentrations. Cell viability was determined after 72 h of exposure to BOLD‐100. One representative of four independent experiments is shown. Statistical significance of differences of IC_50_ values was calculated with one‐way ANOVA with Tukey´s multiple comparisons test: *p<0.05, **p<0.005, ***p<0.001.

The cytoplasmic LD level is a specific marker for the intracellular lipid content.^[^ ^13a^ ^]^ In line with transcriptional data and the enhanced lipid anabolism‐related protein overexpression, LDs were massively enriched in HCTR compared to parental cells (**Figure** **2**a,b). Cellular lipid enrichment was dependent on functional glycolysis since inhibition with glucose analogue 2‐deoxy‐D‐glucose (2‐DG) significantly decreased LD levels only in HCTR cells (Figure 2c). Treatment with BOLD‐100 dose‐ (Figure 2d) and time‐dependently (Figure S2a, Supporting Information) affected Bodipy 493/503 fluorescence intensity only in sensitive HCT116 cells. In detail, short‐term treatment for 6 h led to significant LD increase, indicating a potential initial defense mechanism, while longer treatment durations significantly decreased LD levels in HCT116 cells. In HCTR cells, LD content was stable in the presence of BOLD‐100 (Figure 2d). Relative to the respective corresponding HCT116 data, HCTR cells displayed higher LD levels at every tested time point (Figure S2b, Supporting Information). Surprisingly, in CapanR cells LD levels were significantly lower as compared to parental Capan‐1 cells (Figure S2c, Supporting Information). However, comparable to BOLD‐100‐induced effects observed in the CRC model, Bodipy 493/503 fluorescence intensity was strongly reduced in Capan‐1 cells. The LD‐reducing effect was maximal at 100 µM and even more pronounced than in HCT116 cells. In CapanR cells, only the highest tested concentration (150 µM BOLD‐100) exerted LD‐reducing effects (Figure S2d, Supporting Information). Nonetheless, under treatment, LD levels of CapanR cells were still significantly higher as compared to parental Capan‐1 cells. Taken together, these data strongly support an important role of changes in the lipid compartment potentially determining survival or resistance against BOLD‐100.

### 2.3 Cellular Lipid Content Modulates the Anticancer Activity of BOLD‐100

In a next step, the effects of differing cellular lipid levels on the anticancer activity of BOLD‐100 were assessed. Oleic acid (OA) was used for the induction of intracellular LD levels,^[^ ^31^ ^]^ while the long‐chain fatty acyl CoA synthetase (ACSL) inhibitor triacsin C was utilized to achieve the opposite effect.^[^ ^32^ ^]^ In both tested cell models, addition of OA or triacsin C resulted in an increased or decreased Bodipy 493/503 fluorescence intensity, respectively (Figure 2e). The increase of cellular LD levels with OA pre‐treatment rescued HCT116 cells from BOLD‐100‐induced cytotoxicity (Figure 2f) without affecting intracellular ruthenium accumulation and thus BOLD‐100 uptake (Figure S2e, Supporting Information). Reduction of cellular LD levels by 24 h pre‐treatment with triacsin C induced only an insignificant trend toward enhanced BOLD‐100 responsiveness of HCT116 cells (Figure 2g). In contrast, inhibition of LD formation significantly re‐sensitized HCTR cells for BOLD‐100 as represented by a reduction of the IC_50_ value from 114.1 ± 26.7 µM to 72.6 ± 2.0 µM, rendering the response of HCTR cells toward BOLD‐100 comparable to that of the parental cell line. Contemporaneously, an unbiased screening of BOLD‐100 in combination with 1760 approved drugs was performed with the aim to detect synergistic treatment combinations. In line with the synergizing effect of BOLD‐100 and triacsin C on cell viability reduction, biological process analysis of 129 genes associated with synergistic hits versus 110 antagonistic hits (Figure S3a, Supporting Information) identified links with lipid processing (localization, transport, and metabolic process) as well as regulation of sterol transport (Figure S3b, Supporting Information) in the synergizing responses. Together, these data clearly show a dynamic modulation of the cellular lipid compartment in the immediate response to BOLD‐100 and, furthermore, suggest an amplified anticancer activity of BOLD‐100 upon combination with specific lipid modulators. Additionally, our data strongly support the hypothesis of a protective role of an increased lipid pool contributing to acquired resistance against the ruthenium complex.

### 2.4 The Lipid‐Enriched Phenotype Associated with BOLD‐100 Resistance Generates a Vulnerability toward Lipid Metabolism Inhibitors

**Figure 3:**
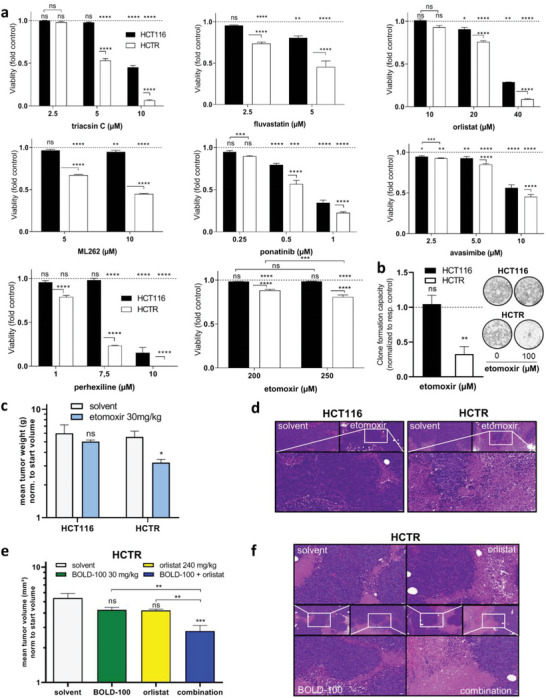
Acquired BOLD‐100‐resistant cells are hypersensitive to inhibitors of multiple steps of the lipid metabolism. a) Cell viability was determined after 72 h of exposure to the indicated drugs at given concentrations (each in triplicates). Statistical significance of differences between HCTR and HCT116 cells was calculated with two‐way ANOVA with Tukey´s multiple comparisons test: *p<0.05, **p<0.005, ***p<0.0005, ****p<0.0001. One representative of three independent experiments delivering comparable results is shown. b) Clone formation capacity of HCT116 and HCTR cells in response to seven days of etomoxir treatment. Two‐way ANOVA with Tukey´s multiple comparisons test: **p<0.005. Representative images are provided on the right. c) Impact of ß‐oxidation inhibition on HCT116 and HCTR xenograft growth. Data on day 27 are shown representatively as means ± standard error of measurement (SEM). Two‐way ANOVA with Sidak´s multiple comparisons test: *p<0.05; n = 4 per experimental group. d) Representative H&E staining of HCT116 and HCTR tumors with respective therapy in xenograft models. Small images: 20x magnification, scale bars: 50 µm; large image: 40x magnification of etomoxir‐treated tumors, scale bar: 20 µm. e) Impact of FASN inhibition via orlistat combined with BOLD‐100 on HCTR xenograft growth. Data on day 28 are shown representatively as means ± SEM. Statistical significance of differences as compared to solvent control group was calculated by one‐way ANOVA with Tukey´s multiple comparisons test and is indicated directly above data bars. Significant differences between individual groups were calculated by unpaired two‐tailed student´s t‐test: **p<0.005, ***p<0.001; n = 4 per experimental group. f) Representative H&E staining of HCTR tumors after indicated therapies in xenograft models.

A wide range of inhibitors affecting different routes of lipid ana‐/catabolism and trafficking was tested to assess if BOLD‐100‐resistant cell survival is dependent on the lipid‐enriched phenotype (**Figure** **3**a). A comprehensive overview on the drug‐respective mode‐of‐action of the utilized inhibitors in the context of intracellular site of execution is provided in Figure S4 (Supporting Information). As compared to parental HCT116 cells, HCTR cells were significantly hypersensitive against triacsin C, the 3‐hydroxy‐3‐methyl‐glutaryl‐CoA reductase (HMGCR) inhibitor fluvastatin targeting the rate‐limiting enzyme of cholesterol synthesis,^[^ ^33^ ^]^ the FASN inhibitor orlistat,^[^ ^34^ ^]^ ML262, a phenylthiophene‐2‐carboxamide inhibiting LD formation in vivo,^[^ ^35^ ^]^ the multi‐tyrosine kinase inhibitor ponatinib recently shown to accumulate in LDs,^[^ ^36^ ^]^ and the acetyl‐CoA acetyltransferase (ACAT) inhibitor avasimibe,^[^ ^37^ ^]^ as well as the carnitine palmitoyl transferase (CPT) inhibitors perhexiline^[^ ^38^ ^]^ and etomoxir.^[^ ^39^ ^]^ Additionally, the clone formation capacity under long‐term treatment with etomoxir was tested (Figure 3b). Only in HCTR cells, prolonged etomoxir treatment significantly reduced clone formation by 67%. These data clearly identify the enhanced lipid anabolism as Achilles' heel of acquired BOLD‐100‐resistant HCT116 cells and, additionally, reveal a dependence on enhanced fatty acid ß‐oxidation. The identified hypersensitivity of HCTR cells to etomoxir prompted us to validate our findings in vivo. We tested single‐drug treatment etomoxir and BOLD‐100 on both HCT116 and HCTR xenograft‐bearing male CB‐17 severe combined immune‐deficient (SCID) mice. Etomoxir therapy significantly reduced HCTR xenograft volume as compared to solvent controls, while HCT116 tumors were widely unaffected (Figure 3c). This is in good agreement with our in vitro hypersensitivity findings (compare Figure 3a). Accordingly, hematoxylin and eosin (H&E) staining showed a reduction of viable tumor regions and an increase in necrotic areas in HCTR tumors treated with etomoxir as compared to solvent controls (Figure 3d). Such alterations were widely undetectable in the parental HCT116 xenografts. Furthermore, as expected, BOLD‐100 significantly reduced xenograft growth of HCT116, but not HCTR tumors (Figure S5a, Supporting Information). This proves persistence of acquired therapy resistance also in vivo. In both treatment regimens, mouse weight was stable over the therapy duration (Figure S5b, Supporting Information). Worth to mention, BOLD‐100 was incubated with mouse serum prior to intravenous (i.v.) injection resulting in a color shift from yellow to green/blue (Figure S5c, Supporting Information). This strategy massively reduced local tail vein reactions (Figure S5d, Supporting Information) observed in previous experiments without the addition of serum and, thus, facilitated i.v. injection and promoted animal well‐being.

In a next step, promising drug combination strategies were evaluated to overcome BOLD‐100 resistance in HCTR cells. Cell viability testing showed, that etomoxir as well as orlistat promoted anticancer activity of BOLD‐100 (Figure S5e,f, Supporting Information). The synergizing effect, however, was more pronounced in the combination with the FASN inhibitor. This prompted us to evaluate combination therapy of orlistat and BOLD‐100 in HCTR xenograft‐bearing SCID mice (Figure 3e). BOLD‐100 and orlistat as single agents only induced an insignificant trend toward tumor reduction. The combination, however, significantly reduced HCTR tumor growth not only as compared to solvent control but also to single agent experimental groups. Histological evaluation of HCTR xenografts confirmed reduced viable tumor regions and increased necrotic areas (Figure 3f).

### 2.5 BOLD‐100 Induces Changes in Mitochondrial Respiration Parameters and Loss of Lactate Transporter Expression

**Figure 4:**
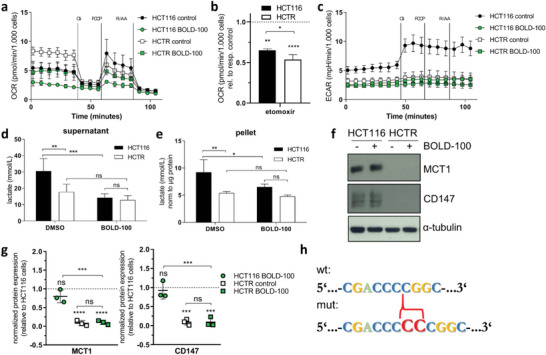
BOLD‐100‐induced mitochondrial respiration changes and lactate secretion loss associated with BSG mutation in HCTR cells. Seahorse FX Pro Mito Stress test measuring a) mitochondrial OCR and c) ECAR of HCT116 and HCTR cells treated with 100 µM of BOLD‐100 or dimethyl sulfoxide (DMSO) for 3 h. Oligomycin (Oli), FCCP, and rotenone/antimycin A (R/A) were added in sequential order with resulting concentrations of 1.5, 1, and 0.5 µM, respectively. b) Quantification of basal OCR after 3 h etomoxir (100 µM) pre‐treatment in HCT116 and HCTR cells. Data are normalized to respective controls. *p<0.05, **p<0.005, ****p<0.0001. d) Extra‐ or e) intra‐cellular lactate secretion or accumulation, respectively, was measured in HCT116 and HCTR cells after treatment with 100 µM of BOLD‐100 or DMSO for 24 h. Two‐way ANOVA with Bonferroni post‐test: *p<0.05, **p<0.005, ***p<0.001; n = 2, each in triplicates. f) Protein expression levels of MCT1 and CD147 in HCT116 and HCTR cells treated with 100 µM of BOLD‐100 or DMSO for 24 h were analyzed by Western blotting. g) Quantification of f normalized to tubulin relative to parental HCT116 cells (dashed line). One‐way ANOVA with Tukey's multiple comparisons test: *p<0.05, **p<0.005; n = 3. h) CC insertion mutation within exon 5 of *BSG* (NM_198589.3:c.539_540dup; red braces) in HCTR cells as detected by WES.

To determine changes in mitochondrial functionality and respiration as well as glycolysis parameters, oxygen consumption rate (OCR) and extracellular acidification rate (ECAR), respectively, were measured. In line with the increased sensitivity toward ß‐oxidation inhibitors and enhanced glucose uptake,^[^ ^21^ ^]^ the basal mitochondrial respiration in HCTR was increased compared to parental HCT116 cells (**Figure** **4**a). However, in both cell models, BOLD‐100 treatment distinctly reduced basal OCR by a comparable amount. Consequently, HCT116 parental cells under BOLD‐100 exposure were widely unresponsive to ATP‐synthase inhibitor oligomycin‐induced OCR reduction. In BOLD‐100‐treated HCTR cells oligomycin still significantly reduced OCR, despite already diminished levels, indicating a higher ATP‐linked respiratory capacity. This persistent respiration most likely derived from lipid enrichment, since HCTR cells were significantly more susceptible to etomoxir‐induced basal OCR reduction (Figure 4b). In HCT116 cells, BOLD‐100 distinctly reduced mitochondrial uncoupler carbonyl cyanide‐p‐trifluoromethoxyphenylhydrazone (FCCP)‐inducible maximal and spare respiratory capacity. Unexpectedly, in HCTR cells maximal inducible respiratory capacity was below basal levels and spare respiratory capacity turned into negative levels. This indicates, that untreated HCTR cells already operate at their bioenergetic limits. In parallel to OCR, ECAR was evaluated as indirect assessment of glycolytic activity through lactate secretion (Figure 4c). BOLD‐100 treatment strongly decreased ECAR only in parental cells. Strikingly, HCTR cells showed massively decreased basal ECAR compared to HCT116 cells. In contrast to untreated HCT116 cells, ECAR of untreated HCTR cells as well as both models under BOLD‐100 treatment became non‐responsive to oxidative phosphorylation inhibition by oligomycin. This is well in line with previously reported defects in glycolysis upon exposure to the ruthenium compound.^[^ ^21^ ^]^ Further analyses showed that the lost lactate secretion (Figure 4d) upon BOLD‐100 treatment and in acquired BOLD‐100 resistance was accompanied by decreased intracellular lactate levels (Figure 4e). Next, we were interested in the mechanisms underlying the distinctly decreased lactate secretion in HCTR cells. This could be explained by strongly reduced expression of lactate transporter MCT1 and its co‐factor CD147 (Figure 4f,g). Relative mRNA expression levels of both the MCT1‐coding *SLC16A1* and the CD147‐coding *BSG gene* were decreased in the resistant cell model (Figure S6a,b, Supporting Information). Moreover, BOLD‐100 treatment decreased *MCT1* mRNA expression in both cell models. However, this effect was more pronounced in HCT116 cells. Vice versa, *BSG* expression was enhanced under treatment. Whole Exome Sequencing (WES) revealed a frameshift‐inducing insertion/duplication mutation (Figure 4h) in exon 5 (*BSG* NM_198 589.3:exon5:c.539_540dup; affected protein CD147 reference (wt) and predicted sequence (mut) Figure S7a,b, Supporting Information) causing a frameshift and translation stop after 70 AA (p.(Gly181Profs*70)). Consequently, protein CD147 wt sequence is changed from position 181 onwards, affecting respectively assigned domains: transmembrane (TM) (position 208–235) and cytoplasmic (position 236–269) region.^[^ ^40^ ^]^ Furthermore, the premature translation stop (at position 249) causes deletion of parts of the cytoplasmic region, which is, together with the TM domain, responsible for the assembly with MCT1.^[^ ^41^ ^]^

### 2.6 BOLD‐100 Treatment Induces Alterations in Histone Acetylation: A Proposed Role of Acetyl‐CoA

**Figure 5:**
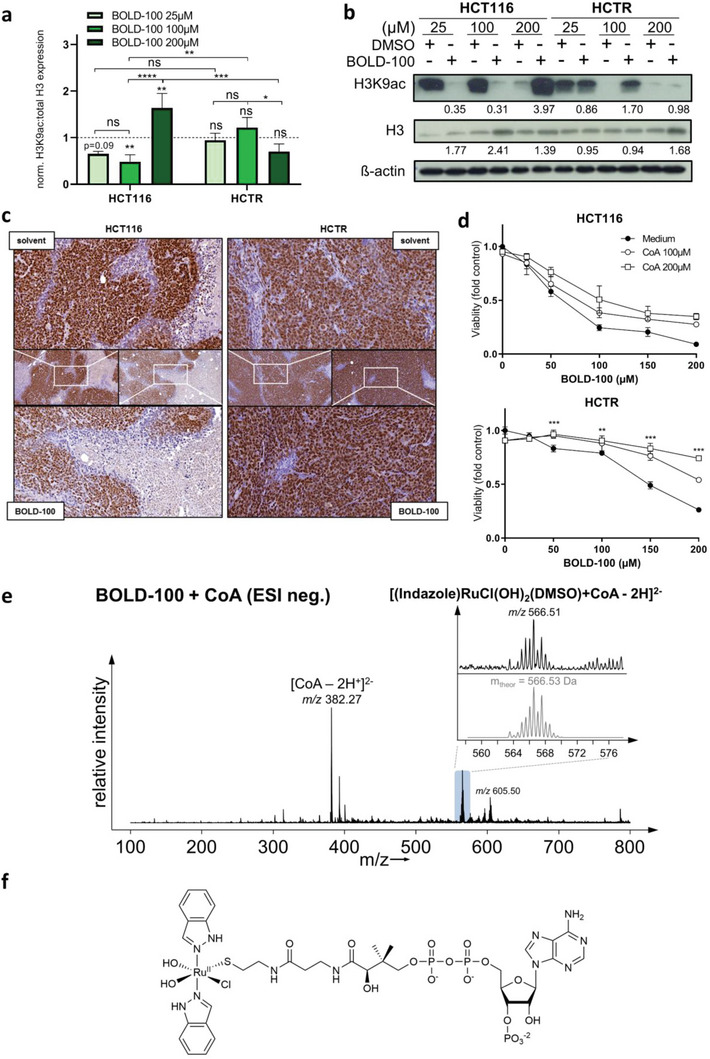
BOLD‐100‐induced epigenetic modification of histone acetylation is accompanied by BOLD‐100‐CoA thiol adduct formation. a) H3K9ac:total H3 protein expression ratio of NE protein fractions of HCT116 and HCTR cells normalized to respective DMSO controls calculated from b. Two‐way ANOVA with Tukey's multiple comparisons test: *p<0.05, **p<0.005, ***p<0.001, ****p<0.0001 b) Expression levels of H3K9ac and H3 in NE protein extracts of HCT116 and HCTR cells treated with indicated doses of BOLD‐100 or DMSO for 24 h analyzed by Western blotting. Numbers below indicate quantified Western blot signal intensities normalized to respective DMSO controls. ß‐actin served as loading control. One representative of three independent experiments is shown. c) IHC analysis of H3K9 acetylation in s.c. HCT116 and HCTR xenografts (micro‐photographs with 20x and 40x magnification). Nuclei and H3K9 acetylation were visualized by hematoxylin (violet) and 3,3′‐diaminobenzidine (brown), respectively. d) Cells were pretreated for 6 h with indicated concentrations of freshly dissolved CoA. BOLD‐100 was added directly into the wells. Cell viability was determined after 72 h of exposure to the indicated drugs at the given concentrations (each in triplicates) using an MTT‐based system. Two‐way ANOVA with Bonferroni post‐test: **p<0.01, ***p<0.001. e) Mass spectra of the interaction between BOLD‐100 and CoA (1:1 molar ratio) acquired in the negative ion mode after 24 h of incubation. f) Proposed structure of a possible BOLD‐100‐CoA reaction product identified by NMR analysis.

Since both ß‐oxidation as well as the TCA cycle produce acetyl‐CoA, the key cellular acetyl‐group donor for histone acetylation,^[^ ^27^ ^]^ we tested a potential epigenetic impact of BOLD‐100 treatment (**Figure** **5**a,b). Analysis of nuclear‐enriched (NE) protein fractions revealed a dose‐dependent decrease of H3K9ac and an increase in total H3 in HCT116 cells. The strong increase of H3K9ac together with H3 expression levels in HCT116 cells upon treatment with 200 µM BOLD‐100 are probably a cellular stress response due to the deadly concentration of the compound. In HCTR cells under BOLD‐100 treatment, H3K9ac was only marginally affected. Correspondingly, immunohistochemical (IHC) evaluation of H3K9ac in HCT116 and HCTR xenografts revealed reduction of the epigenetic mark by BOLD‐100 treatment specifically in sensitive tumors (Figure 5c). Based on the fact that acetyl‐CoA represents the major substrate for histone acetylation,^[^ ^27^, ^28^ ^]^ together with the observed loss of histone acetylation in the sensitive cell model, we postulated a potential interaction between BOLD‐100 and CoA. Indeed, co‐incubation of BOLD‐100 with increasing concentrations of CoA progressively abolished BOLD‐100 in vitro anticancer activity even in the presence of serum‐derived albumin known to bind BOLD‐100 (Figure 5d).^[^ ^7^ ^]^ Electron Spray Ionization (ESI)‐ mass spectrometry (MS) of either BOLD‐100 or etomoxir in the presence of CoA was performed under cell‐free conditions. Etomoxir is known to covalently bind to CoA via nucleophilic addition forming a thioester^[^ ^42^ ^]^ and, thus, served as a positive control. In our analyses, etomoxir‐CoA‐binding could be confirmed, verifying appropriate measurement conditions (Figure S8a, Supporting Information). Concerning BOLD‐100, ESI‐MS indeed revealed a specific reaction between BOLD‐100 and CoA under cell‐free conditions (Figure 5e). The reaction products were found in the presence and absence of the mild reductant dithiothreitol and were tentatively assigned to [(Indazole)RuCl(OH)_2_(DMSO)+CoA‐2H]^2−^ (m/z 566.51, m_theor_ = 566.53). For further characterization and to exclude potential electrospray artefacts, the reaction of BOLD‐100 and CoA was analyzed by nuclear magnetic resonance (NMR) (Figure S8b, Supporting Information). The NMR signals 1–3 of the nucleobase region of CoA revealed a specific reaction between CoA and BOLD‐100 with a 50% conversion rate after 6 h and completion after 16 h. Paramagnetic indazole‐Ru(III) signals disappeared, and indazole‐Ru(II) peaks were found between 7–8 ppm. CH_2_‐signals at position 12 and 13 adjacent to the thiol group of CoA shifted down‐field indicative of a potential coordination of BOLD‐100 with CoA at the thiol group or N7 of adenosine in a chelating manner. The reaction was accompanied by the characteristic color shift from orange to yellow‐green (Figure S8c, Supporting Information) observed already previously upon co‐incubation with serum (compare Figure S5c, Supporting Information) and was also reported from patient i.v. injections. Based on these data, we propose the BOLD‐100‐CoA thiol adduct as a reaction product of relevance (Figure 5f). BOLD‐100‐CoA adduct formation gives one reasonable explanation for the limited availability of acetyl‐groups for histone modification in BOLD‐100‐treated HCT116 cells. Fittingly, analysis of gene expression data using the gene ontology enrichment analysis and visualization tools (GOrilla) (Figure S9a,b, Supporting Information) identified “NUCLEOSOME ASSEMBLY”, “PROTEIN‐DNA‐COMPLEX ASSEMBLY”, “NUCLEOSOME ORGANIZATION”, and “PROTEIN‐DNA COMPLEX SUBUNIT ORGANIZATION” as the top four enriched GO terms upon BOLD‐100 treatment as compared to untreated HCT116 cells. In Capan‐1 cells, the same GO terms were among the top twenty enriched ones upon BOLD‐100 treatment, corresponding well to the regulations in the CRC model and implying a general relevance for the mode‐of‐action of BOLD‐100.

### 2.7 Histone Gene Expression Predicts Intrinsic BOLD‐100 Responsiveness

Acetylation of histone gene promoters has been reported as central regulatory system for histone gene expression.^[^ ^43^ ^]^ Consequently, we re‐analyzed whole genome gene expression patterns of intrinsically BOLD‐100‐resistant CCL13 and SW480 cells in comparison to sensitive HCT116 and Capan‐1 cells without and with BOLD‐100 treatment.^[^ ^1a^ ^]^ In sensitive models, BOLD‐100 treatment massively upregulated multiple histone‐associated gene sets, while in intrinsically resistant models the gene set “RIBOSOME” was top enriched (Figure S10a, Supporting Information), and changes of histone‐associated gene sets were of minor importance. Accordingly, histone‐related gene sets also dominated the comparison of untreated intrinsically resistant versus sensitive models (Figure S10b, Supporting Information), with intrinsically sensitive cells expressing lower levels of multiple histone genes. Especially, expression of all core histone gene families (H2, H3, and H4) was massively upregulated following 6 h BOLD‐100 treatment (Figure S10c, Supporting Information). However, in BOLD‐100‐resistant SW480 and CCL13 cells under treatment the GO‐terms “HISTONE MODIFICATION” (*p*‐value = 1.96E^−8^, FDR *q*‐value = 3.18E^−6^) and “HISTONE ACETYLATION” (*p*‐value = 3.78E^−5^, FDR *q*‐value = 3.34E^−3^) were significantly enriched, containing among others the H3 lysine 9‐associated HAT *KAT6A* gene.^[^ ^44^ ^]^ In line with the regulation pattern of H3K9 acetylation (compare Figure 5b,c), relative *KAT6A* mRNA levels were decreased in HCT116, but further increased in HCTR cells under BOLD‐100 exposure (Figure S10d, Supporting Information).

### 2.8 Potential Factors Stabilizing Histone Acetylation in HCTR Cells under BOLD‐100 Treatment

**Figure 6:**
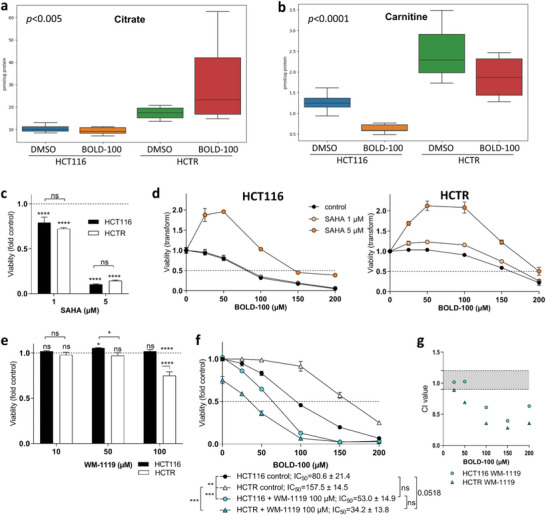
Histone acetylation as regulator of BOLD‐100 resistance. Impact of BOLD‐100 (24 h, 100 µM) or solvent control DMSO exposure on the level of a) citrate and b) carnitine in HCT116 and HCTR cells determined by metabolomics. All metabolites are given in pmol/µg protein, *n* = 6 biological replicates. Cell viability was determined after 72 h of treatment with c) HDAC inhibitor SAHA, and e) KAT6A inhibitor WM‐1119 as single drugs or as combination of BOLD‐100 with d) SAHA or f) WM‐1119 at the indicated concentrations. One representative of three independent experiments is shown each. Statistical significance of differences was tested using two‐way ANOVA with Tukey´s multiple comparisons test: *p<0.05, **p<0.005, *** *p*<0.001**** *p*<0.0001. g) Combination index (CI) values were calculated for the dose‐effect curves in f.

Considering BOLD‐100–CoA adduct formation, the question arose how HCTR cells maintain histone acetylation under acute BOLD‐100 treatment. Metabolomic analyses showed an insignificant but obvious trend toward depletion of intracellular acetyl‐CoA levels in HCT116 but not HCTR cells (Figure S11, Supporting Information). Citrate was significantly upregulated in the acquired resistance model without and with acute BOLD‐100 treatment (**Figure** **6**a). Moreover, the ß‐oxidation substrate carnitine level was increased in the resistance model (Figure 6b), supporting the above discussed switch toward enhanced dependence on ß‐oxidation and highlighting another route potentially fueling enhanced histone acetylation in BOLD‐100 resistance. To assess the role of histone modification mediating BOLD‐100 resistance, several inhibitors targeting histone‐modifying enzymatic activity were utilized. Vorinostat (SAHA) is an inhibitor of class I, II, and IV histone deacetylases (HDAC), thus promoting histone acetylation.^[^ ^45^ ^]^ In cell viability assays, HCT116 and HCTR displayed comparable sensitivity to SAHA alone (Figure 6c). In combination experiments, BOLD‐100 clearly antagonized SAHA anticancer activity in both cell models (Figure 6d). This supports an opposing impact of the two compounds on histone acetylation. This assumption is also supported by the above‐described enhanced *KAT6A* mRNA expression levels in HCTR cells (compare Figure S10d, Supporting Information). To challenge this hypothesis, cell viability was assessed under treatment with KAT6A inhibitor WM‐1119.^[^ ^46^ ^]^ Indeed, HCTR cells were hypersensitive to selective KAT6A inhibition (Figure 6e). Moreover, combination with WM‐1119 enhanced BOLD‐100 activity especially in the HCTR model and completely reverted acquired resistance to the ruthenium complex (Figure 6f,g).

## 3 Discussion

**Figure 7:**
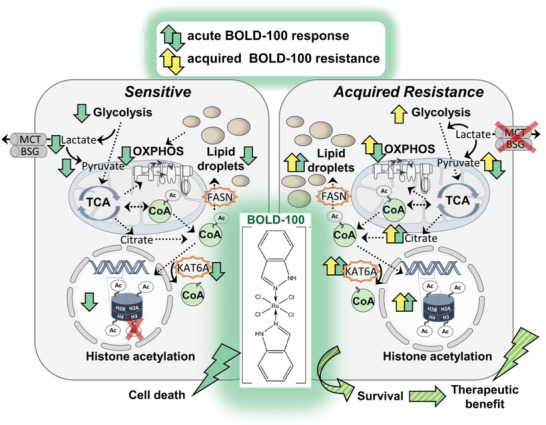
Summary of identified regulated metabolic pathways. In sensitive parental HCT116 cells, the anti‐Warburg compound BOLD‐100 reduces substrates of TCA cycle and ß‐oxidation (lactate, pyruvate, and LDs), consequently, reducing histone acetyl group donor acetyl‐CoA, associated with BOLD‐100‐CoA adduct formation. Acquired BOLD‐100‐resistant cells combat (BOLD‐100‐induced) metabolic exhaustion via upregulation of onco‐metabolic pathways including glycolysis, LD formation, ß‐oxidation, and OXPHOS contributing to stable histone acetylation. Citrate production is additionally fueled by mutation of BSG mutually destabilizing lactate exporter MCT expression in HCTR cells. Short and long‐time exposure to BOLD‐100 deregulates KAT6A activity rendering HCTR cells proficient of histone‐acetylating enzymatic activity promoting cellular survival even in presence of BOLD‐100. Identification of augmented dependence on glycolysis, fatty acid anabolism, ß‐oxidation, as well as KAT6A activity allows for specific targeting of metabolic routes driving resistance in order to re‐sensitize against BOLD‐100.

In the present study, we focused on the dissection and characterization of the mode‐of‐action of BOLD‐100 as well as mechanisms underlying resistance against this clinically investigated ruthenium complex. This is of central importance, since mechanisms involved in ruthenium‐drug resistance remain widely enigmatic so far, which might cause drawbacks in their efficient development for clinical use.^[^ ^3^ ^]^ A scheme providing a comprehensive summary about the central metabolic pathways identified to be regulated by BOLD‐100 in this study is given in **Figure** **7**.

Intrinsically BOLD‐100‐sensitive CRC HCT116 and pancreatic cancer Capan‐1 cell lines were chosen to establish acquired BOLD‐100‐resistant sublines based on previously executed BOLD‐100 sensitivity screens.^[^ ^1a^ ^]^ Additionally, from these screens SW480 and CCL13 cells were selected as intrinsically BOLD‐100‐resistant models. Here, we uncover a strong impact of BOLD‐100 on the cellular lipid metabolism and reveal regulation of BOLD‐100 anticancer activity by the lipid compartment. Precisely, cellular neutral lipid storage organelles of HCT116 and Capan‐1 cells were dynamically regulated by BOLD‐100 in a dose‐ and time‐dependent manner. The observed LD‐inducing short‐time treatment effect of BOLD‐100 was supported by earlier observations of LD induction after treatment up to 6 h with the BOLD‐100 precursor KP1019 in yeast and HeLa cells.^[^ ^47^ ^]^ Previous studies described the cellular lipid metabolism as determining factor of platinum‐based therapy efficacy and in the development of therapy resistance.^[^ ^6^ ^]^ Moreover, we recently demonstrated that metabolic reprogramming associated with acquired BOLD‐100 resistance in HCT116 cells distinctly differs from oxaliplatin resistance through, among others, a more extensive reprogramming of the fatty acid metabolism.^[^ ^20^ ^]^ In BOLD‐100‐resistant compared to parental HCT116 cells, transcriptional and translational changes in the lipid biosynthesis machinery increased *de novo* lipid production. Lipid metabolism in CapanR cells, in contrast, was characterized by decreased lipid pools associated with reduced *FASN* and *DGAT2* but enhanced lipid transporter *CD36* gene expression. Independent of basal lipid contents, acute BOLD‐100 treatment significantly reduced lipid stores in both parental cell models. In contrast, the resistant sublines preserved their LD levels under BOLD‐100 exposure. The recently reported enhanced glycolytic activity of the BOLD‐100‐resistant CRC model^[^ ^21^ ^]^ significantly contributes to the immense accumulation of LDs in the HCT116 model. In addition, we demonstrated that these metabolic shifts culminate in a lipid‐dependent phenotype. This survival dependency on the lipid surplus rendered BOLD‐100‐resistant cancer cells stably hypersensitive to a wide range of lipid anabolism inhibitors, providing a rationale to fight acquired BOLD‐100 resistance. Accordingly, pharmacologic reduction of fatty acids and LDs reversed acquired BOLD‐100 resistance. Lately, a novel ruthenium‐fluvastatin complex showed activity against mammary carcinoma in rodents.^[^ ^48^ ^]^ This study by Liang et al. presented a promising strategy for therapy combination approaches, but did not address the effects of the ruthenium chloride‐fluvastatin complex on ruthenium‐based therapy resistance. By combining BOLD‐100 with orlistat in HCTR xenograft models, we demonstrated that inhibition of FASN reverted acquired resistance, translating our findings to the in vivo situation and supporting a potential clinical relevance of this therapeutic approach. Nonetheless, further investigations are needed to develop the optimized combination strategy with onco‐metabolism‐targeting compounds to overcome acquired BOLD‐100 resistance.

Besides enhanced lipid levels, a diminished lactate secretion – determined by a biochemical assay and ECAR in Seahorse experiments – accompanied by loss of MCT1 expression characterized HCT116 cells with acquired BOLD‐100 resistance. To the best of our knowledge, MCT1 expression loss associated with acquired metal‐based chemotherapy resistance is unprecedented until today. Generally, lactic acid is excreted by cancer cells to maintain intracellular pH balance,^[^ ^49^ ^]^ but might also be reconverted to pyruvate (as observed in “reverse Warburg effect”) for fueling of the TCA cycle.^[^ ^50^ ^]^ In this case, reduced ECAR is obviously not indicating reduced aerobic glycolysis, as routinely assumed in standard Seahorse assays, but caused by blocked lactate excretion due to MCT1 loss. Lambert et al. discovered a significant decrease in MCT1 protein expression in colonic epithelia in the process of malignant transformation and suggested a switch from butyrate toward glucose as an energy source.^[^ ^51^ ^]^ This corroborates our previously published observation of enhanced glycolysis and dependency on glucose in HCTR cells.^[^ ^21^ ^]^ The distinctly increased pyruvate but decreased intracellular lactate levels in the resistant subline suggest a preferential fueling into the TCA cycle supporting mitochondrial respiration, well in agreement with distinctly enhanced OCR. Previous publications point out a clear interdependency of MCTs and their important chaperone CD147 co‐affecting MCT expression,^[^ ^52^ ^]^ maturation,^[^ ^53^ ^]^ transport to the plasma membrane,^[^ ^24^, ^25^ ^]^ and turnover.^[^ ^25b^ ^]^ Absence of MCT1 in total protein extracts, but only marginally reduced gene transcription in mRNA analyses suggests, that the protein is either not translated or highly unstable in HCTR as compared to HCT116 cells. In support of the second hypothesis, we detected a frameshift‐inducing insertion mutation in the CD147‐coding *BSG* gene, depleting the TM and cytoplasmic region, domains required for assembly with MCT1.^[^ ^41^ ^]^ The massive CD147 loss yields one sound explanation for the strongly decreased MCT1 expression.^[^ ^25^ ^b,^ ^53^, ^54^ ^]^ The functional background for this MCT1 loss in HCTR cells might lie in the need to use lactate as metabolic fuel. Thus, analyses of lactate levels revealed not only a loss of secretion but also reduced intracellular accumulation. This, supported by the enhanced pyruvate pool^[^ ^21^ ^]^ and the LD enrichment associated with BOLD‐100 resistance, argues for the theory that HCTR cells increase their use of lactate as metabolic building block. As extracellular lactate exerts massive immunosuppressive functions^[^ ^55^ ^]^ and BOLD‐100 induces characteristics of immunogenic cell death in colon cancer spheroids,^[^ ^56^ ^]^ we currently investigate, in how far a respective metabolism‐based, immune‐stimulatory effect might contribute to the in vivo anticancer effects of this ruthenium complex.

The anti‐Warburg activity of acute BOLD‐100 treatment,^[^ ^21^ ^]^ together with depletion of the lipid storage compartment and, vice versa, enhanced glycolysis and lipid anabolism in the resistant HCT116 subline, strongly support that the mode‐of‐action of BOLD‐100 includes distinct metabolic bottle‐neck induction. Both metabolic processes, glycolysis/TCA cycle and ß‐oxidation, produce, besides redox equivalents for oxidative phosphorylation, the specific protein acetylation substrate acetyl‐CoA. Consequently, we wondered, whether acetyl‐CoA and subsequent histone acetylation might be targeted by BOLD‐100. Indeed, a spontaneous BOLD‐100‐CoA adduct formation was proven under cell‐free conditions, delivering one possible explanation for the loss of histone acetylation observed in BOLD‐100‐treated HCT116 cells, exemplarily shown for the activating H3K9ac mark. In HCTR cells, in contrast, histone acetylation was even increased upon BOLD‐100 treatment potentially fueled by both, increased glycolytic activity (with enhanced pyruvate levels), and utilization of the enhanced lipid stores via ß‐oxidation. In support of this hypothesis, the H3K9‐specific HAT *KAT6A* mRNA expression regulation paralleled the one of H3K9 acetylation in HCT116 and HCTR models without and with BOLD‐100 exposure. Accordingly, HCTR cells were more sensitive to KAT6A inhibition alone, and combination with BOLD‐100 synergistically reduced cell viability and completely reverted acquired BOLD‐100 resistance. This, together with the antagonism with HDAC inhibitor SAHA, supports a central role of histone deacetylation in the mode‐of‐action of BOLD‐100. Corroborating, metabolomics showed upregulation of several onco‐metabolites, representative of metabolic pathways including glycolysis, LD formation, and ß‐oxidation, as a consequence of BOLD‐100 resistance, contributing to stable histone acetylation. Nonetheless, how sensing of the metabolic drainage upon long and short‐term exposure toward BOLD‐100 is mediated intracellularly remains an open question and is matter of ongoing investigations. Studies on isolated yeast nucleosomes suggested spatial assembly of the BOLD‐100 precursor KP1019 with H3 and H4, thereby regulating chromatin architecture and nucleosome stability.^[^ ^47^, ^57^ ^]^ Furthermore, yeast knockout clones of multiple histone acetylation‐associated genes were characterized by enhanced responsiveness toward KP1019 with the strongest effect observed for H3K9‐, H3K27‐, and H3K56‐associated HAT rtt109. The authors conclude that these histone acetylation processes protect against KP1019 DNA damage stress in yeast. Accordingly, H3K9 acetylation modification correlated with BOLD‐100 anticancer activity in our human tumor cell models. Our data add a new layer of complexity onto this interaction by demonstrating direct regulation of histone acetylation by BOLD‐100, depending on the resistance phenotype. The reduction of the key HAT substrate acetyl‐CoA as a molecular mechanism of BOLD‐100‐mediated gene expression deregulation was strongly supported by a plethora of our metabolic analyses. Furthermore, supporting this conclusion, a transcriptomic analysis of two intrinsically BOLD‐100‐sensitive versus –resistant cancer cell models indicated a core histone gene expression response as central predictive biomarker for BOLD‐100 activity. Histone gene expression is regulated in a complex manner also by histone acetylation processes,^[^ ^43^ ^]^ but also by the central metabolite pyruvate even when added extracellularly.^[^ ^58^ ^]^ In how far the massive pyruvate depletion in BOLD‐100 responsive cells^[^ ^21^ ^]^ is underlying the strong activation of histone gene expression in response to acute BOLD‐100 treatment is matter of ongoing investigations.

Summarizing, we here present the ruthenium complex BOLD‐100 as an epigenetic gene expression modulator based on a complex interference with the onco‐metabolome. Whether metabolic reduction of protein acetylation is underlying also other modes‐of‐action reported for BOLD‐100 before needs to be determined in further analyses. In that respect, it is interesting to mention, that cellular GRP78 processing, considered as major player in BOLD‐100 activity^[^ ^1^ ^a,^ ^8^ ^]^ is at least in part regulated by acetylation of this central ER stress chaperon.^[^ ^59^ ^]^

## 4 Conclusion

In conclusion, we identify the first‐in‐class anticancer ruthenium compound BOLD‐100 as epigenetically active substance targeting several onco‐metabolic pathways. Enhanced *de novo* lipid synthesis, based on increased glycolytic activity, and increased dependence on ß‐oxidation were uncovered as vital and targetable players in acquired therapy resistance. BOLD‐100 significantly reduced production and release of lactate, a major immunosuppressive metabolite. Interference with the onco‐metabolome, associated with direct BOLD‐100‐CoA adduct formation, cross‐talked to epigenetic gene expression regulation via inhibition of histone acetylation. Our findings will not only distinctly impact the perception of this lead anticancer ruthenium compound, currently on the edge to clinical application, but may also open new perspectives in metal‐drug research in general.

## 5 Experimental Section

### Chemicals, Compounds

BOLD‐100 was obtained from Bold Therapeutics Inc. (Vancouver, Canada), dissolved in DMSO to a 20 mM stock solution, and further diluted to respective concentrations using complete medium. Triacsin C was purchased from Biomol GmbH (Hamburg, Germany). Fluvastatin sodium salt hydrate was purchased from TCI Deutschland GmbH (Eschborn, Germany). Orlistat was a generous gift from Prof. C. Kowol (University of Vienna, Vienna, Austria). Ponatinib and avasimibe were purchased from Selleckchem (Munich, Germany). Etomoxir was obtained from Adooq Bioscience LLC (A11415, Irvine, CA, USA). ML262 was purchased from Glixx Laboratories Inc (GLXC‐02254, Hopkinton, MA, USA). (+)‐Etomoxir sodium salt hydrate (E1905), perhexilin maleate salt (SML0120), CoA (C4780), and bovine serum albumin (BSA)‐conjugated oleic acid (03008) as well as 2‐DG were purchased from Sigma–Aldrich (St. Louis, MO, USA). D‐(+)‐Glucose monohydrate was obtained from Merck KGaA (108342, Darmstadt, Germany). WM‐1119 was bought from THP Medical Products (Vienna, Austria). SAHA was obtained from LC Labs (Woburn, MA, USA).

### Cell Culture

Human CRC HCT116 cells were generously provided by Dr. Vogelstein (John Hopkins University, Baltimore). Human pancreatic adenocarcinoma Capan‐1, CRC SW480, and hepatoma CCL13 cells were obtained from the American Type Culture Collection (ATCC, Manassas, VA, USA). HCT116 cells were cultured in McCoy's 5A Modified Media (Sigma–Aldrich, St. Louis, MO, USA) supplemented with 10% heat‐inactivated fetal calf serum (FCS, Biowest, Nuaillé, France), and 2 mM glutamine (Sigma–Aldrich, St. Louis, MO, USA). Capan‐1 and CCL13 cells were grown in RPMI‐1640 (Sigma–Aldrich, St. Louis, MO, USA) supplemented with 10% FCS. SW480 cells were cultivated in Minimum Essential Medium Eagle medium (Sigma–Aldrich, St. Louis, MO, USA) supplemented with 10% FCS. HCTR and CapanR sublines were generated as described before.^[^ ^21^ ^]^ In brief, cells were selected for acquired BOLD‐100 resistance via intermittent exposure to increasing concentrations up to 200 µM of the ruthenium compound. Cell cultures were grown under 5% CO_2_ atmosphere at 37 °C and regularly checked for contamination with *Mycoplasma*.

### Cell Viability Assays

HCT116 and HCTR cells were seeded at a density of 3.5 × 10^4^ cells mL^−1^ in 96 well plates in 100 µL well^−1^ of cell culture medium and allowed to adhere overnight. Cells were exposed to indicated concentrations of the respective drugs or compounds for indicated time periods. For respective experiments, cellular LDs were increased by 72 h treatment with OA prior to drug exposure. For co‐incubation experiments, CoA was dissolved freshly in 0.9% NaCl and cells were immediately treated to avoid oxidation of the compound. BOLD‐100 was added directly into the wells after 6 h of pre‐incubation with CoA and cells were incubated for 72 h. Cell viability was determined using the MTT assay (EZ4U, Biomedica, Vienna, Austria) following manufacturer's recommendations. Colorimetric signals were measured spectrophotometrically at 450 nm and a reference wavelength of 620 nm using the Asys expert plus micro plate reader (v1.4, Asys Hitech GmbH Hitech, Eugendorf, Austria). Data were analyzed using GraphPad Prism software (v8.0.1, La Jolla, CA, USA). Half‐maximal inhibitory drug concentrations, IC_50_ values, were calculated from full dose‐response curves by non‐linear regression curve‐fitting (sigmoidal dose‐response with variable slope). CI values were calculated by the method described by Chou and Talalay.^[^ ^60^ ^]^

### Clone Formation Assays

For clonogenic assay, HCT116 and HCTR cells were seeded as 2000 cells mL^−1^ in 250 µL cell culture medium in 24‐well microtiter plates. The following day, cells were treated with 100 µM of etomoxir for 7 days. Cells were washed with ice‐cold phosphate‐buffered saline (PBS, pH 7.4 Sigma–Aldrich Inc., St. Louis, MO, USA), fixed, washed, and stained with 0.01% (w/v) crystal violet dissolved in ethanol for 5 min. Following, washed and dried plates were scanned for fluorescent signal (610/30 nm BP emission filter, 633 laser) of stained cell clones using a Typhoon TRIO Variable Mode Manager Imager System (Amersham 168 Bioscienes, UK) with the Typhoon Scanner Control software (V5.0). Pixel intensities per well were quantified by Image J 1.50i (NIH, USA).

### Cellular Ruthenium Uptake Measurements

To determine the intracellular ruthenium accumulation, HCT116 and HCTR cells with or without 72 h pre‐treatment with OA were seeded at a density of 1 × 10^5^ cells in triplicates in 6‐well plates in 2 mL of cell culture medium and allowed to adhere overnight. Cells were treated with 100 µM of BOLD‐100 for 24 h. To measure the absorption of the compound to the plastic well, BOLD‐100 was additionally added to cell‐free blank wells. Processing of samples, measurement of intracellular ruthenium content, and data evaluation were performed as described before.^[^ ^61^ ^]^ In brief, cells were washed twice with PBS, lysed in 500 µL of 0.6 N HNO_3_ (67%, NORMATOM, VWR International, LLC, Belgium) at room temperature (RT) for 1 h. The resulting lysates were filled up to a total volume of 8 mL with bidistilled H_2_O. A quadrupole‐based inductively coupled plasmon mass spectrometry (ICP‐MS) instrument Agilent 7800 (Agilent Technologies, Tokyo, Japan) equipped with the Agilent SPS 4 autosampler (Agilent Tech‐nologies, Tokyo, Japan) and a MicroMist nebulizer at a sample uptake rate of ≈0.2 mL min^−1^ was utilized to measure the total cellular ruthenium content. ^102^Ru standards were derived from Labkings (Hilversum, The Netherlands). Argon was applied as plasma gas (15 L min^−1^) and carrier gas (1.06 L min^−1^). The integration time was set to 0.3 s with 10 replicates and 100 sweeps/replicate. As internal standard for ruthenium, ^115^In was deployed. Data evaluation was performed with the Agilent MassHunter software package (Workstation Software, Version C.01.04, 2018). Data were normalized to respective cell numbers.

### Total‐RNA Isolation and Whole Genome Gene Expression Analyses

Whole genome gene expression arrays were performed as described before.^[^ ^62^ ^]^ In brief, mRNA was isolated from HCT116 and Capan‐1 cells, their respective acquired BOLD‐100‐resistant sublines, as well as intrinsically BOLD‐100‐resistant SW480 and CCL13 cells using the RNeasy Mini Kit (QIAGEN GmbH, Hilden, Germany) according to manufacturer's recommendation. Whole genome gene expression analyses were performed on 4 × 44 K oligonucleotide‐based microarrays (G4845A, Agilent Technologies, Santa Clara, CA, USA) according to manufacturer's instructions. Feature extraction was carried out using the Feature Extraction software (version 11.5.1.1.). Differentially expressed genes in BOLD‐100‐resistant versus respective parental cells or parental cells and intrinsically resistant models with or without treatment with 100 µM BOLD‐100 for 6 h were analyzed using the GeneSpring software (version 13.0). GSEA on the loess (within arrays) and quantile (between arrays) normalized gene expression matrix was done on the C2 dataset of the molecular signature database (version 7.4) using the GSEA software (version 4.0.3) with default parameters and the log_2_FC as ranking metric.^[^ ^63^ ^]^

### Gene Ontology Enrichment Analysis and Visualization Tool

Whole genome gene expression data were analyzed using the GOrilla tool published by Eden et al.^[^ ^64^ ^]^ mRNA expression data were ranked by logFC and tested against the gene ontology “biological process” with the ranked dataset as test set and the entirety of genes annotated by any term of the gene‐ontology “biological process” as reference set. Significantly enriched GO‐terms were visualized as an acyclic tree with GO‐terms as nodes colored according to their respective *p*‐values.

### Biological Process Analyses

HCT116 cells (ATCC®: CCL‐247) were cultivated in McCoy 5A medium (Wisent, St‐Bruno, Qc, CA) supplemented with 10% Premium fetal bovine serum (FBS, Wisent, St‐Bruno, Qc, CA) in a humidified incubator (37 °C, 5% CO_2_). HCT116 cells were seeded at a density of 3.5 × 10^4^ cells mL^−1^ in 96‐well plates in 100 µL well^−1^. On the next day, cells were treated with 100 µM of BOLD‐100 or in simultaneous combination with Pharmakon library (1760 FDA and EMA approved drugs) at 10 µM final concentration (MSdiscovery, Gaylordsville, CT, USA) for 72 h. After treatment, media were collected, and cells were rinsed with PBS (Wisent, St‐Bruno, Qc, CA), detached with trypsin (ThermoFisher Scientific, Waltham, MA, USA) for 7 min, re‐supsended in their original media (containing dead cells). Cell viability was measured using Viacount staining dye (Luminex, Austin, TX, USA) according to manufacturer's specification prior to data acquisition using Guava flow cytometer (Millipore EMD/Sigma, Guava EasyCyte 6HT 2L). Cell viability in response to BOLD‐100 and the respective combination was expressed relative to vehicle treated cells. Drug combination effects were determined by calculating synergy indexes as previously reported.^[^ ^65^ ^]^ To define the molecular targets and pathways involved in the synergistic and antagonistic responses with BOLD‐100, “Drug Gene Interaction Database” (DGIdb, https://www.dgidb.org) was used. Two gene lists corresponding to antagonistic and synergistic drugs were generated. Subsequently, GOrilla tool was utilized to identify enriched GO terms, in particular with the "biological process" term, that are associated with antagonistic and synergistic interactions between BOLD‐100 and 1760 approved drugs.

### WES

DNA was isolated from HCT116 and HCTR cells using QIAamp DNA Blood Mini Kit (Qiagen, Vienna, Austria) according to manufacturer's recommendations. Quantity of DNA was assessed by Qubit Fluorometric Quantitation system (Life Technologies, Carlsbad, CA, USA). Libraries were prepared by using the Twist Library Preparation EF Kit (Twist Bioscience, South San Francisco, CA, USA). Briefly, genomic DNA was fragmented, size‐selected and amplified, followed by hybridization with biotinylated baits and capture with streptavidin‐conjugated magnetic beads. After enrichment, library fragments representing in total 37Mb coding region (Twist Human Core Exome + RefSeq Panel) were amplified and size‐selected. Final library pools were quality controlled using Qubit Fluorometric Quantitation system (Life Technologies, Carlsbad, CA, USA) and automated electrophoresis Bioanalyzer system (Agilent Technologies, Tokyo, Japan). Libraries were sequenced on a NovaSeq 6000 instrument (Illumina, San Diego, CA, USA) using 100 bp paired‐end chemistry. All library preparation and sequencing steps were performed by the Biomedical Sequencing Facility at the Center of Molecular Medicine (CeMM, Vienna, Austria). Sequences were then analyzed at the Neuromuscular Research Department using an in‐house developed bioinformatics pipeline starting from two separate bam files per sample containing unaligned reads. First, bam files were converted to fastq files using BEDTools (version 2.30)^[^ ^66^ ^]^ and merged to single sets of matched paired‐end reads. For mapping to the human genome reference sequence (humanG1Kv37 based on GRCh37), the BWA‐MEM algorithm (version 0.7.14) was used.^[^ ^67^ ^]^ Marking of duplicate reads was performed with SAMBLASTER (version 0.1.26)^[^ ^68^ ^]^ and conversion to sorted bam files containing aligned reads was performed using Sambamba (version 0.8.2).^[^ ^69^ ^]^ Next, bam files were re‐calibrated using the Genome Analysis Tool Kit (GATK, version 3.8)^[^ ^70^ ^]^ before variant calling was performed using either the GATK Haplotype Caller or the Mutect2 algorithm in GATK version 4.2.5.0. Finally, variant annotation was done using ANNOVAR^[^ ^71^ ^]^ and exported to Excel spreadsheets for final analysis. Raw data were submitted to the European Bioinformatics Institute (EMBL‐EBI, Cambridgeshire, UK).

### Western Blot Analyses

For protein expression analysis from whole cell lysates of HCT116 and HCTR cells, 0.7 × 10^6^ cells were seeded in 6‐well plates in 2 mL of cell culture medium and allowed to adhere overnight. For NE protein fractions, 3 × 10^6^ cells were seeded in T75 flasks in 10 mL of cell culture medium and allowed to adhere overnight. Cells were left untreated for whole cell protein isolation or treated with the indicated concentrations of BOLD‐100 or the corresponding amounts of DMSO for 24 h in case of NE preparation. Samples for whole cell protein analysis were collected and extracted as described before.^[^ ^72^ ^]^ For NE preparation the NE‐PER™ Nuclear and Cytoplasmic Extraction Kit (78 833, Thermo Fisher Scientific, Waltham, MA, USA) was used according to manufacturer's recommendations. The Micro BCA™ Protein Assay Kit (Thermo Fisher Scientific, Waltham, MA, USA) was used to determine protein concentrations of whole cell or NE lysates. For the separation of proteins, sodium dodecyl sulfate polyacrylamide gel electrophoresis (SDS‐PAGE) was performed. Following, separated proteins were transferred onto polyvinylidene difluo‐ride membranes (PVDF, Thermo Fisher Scientific, Waltham, MA, USA). Primary antibodies anti‐FASN (A‐5) (sc‐55580, dilution 1:1000), anti‐DGAT1 (A5) (sc‐271934, 1:200), anti‐MCT1 (H‐1) (sc‐365501, 1:250), and anti‐CD147 (8D6) (Emmprin, sc‐21746, 1:1000) were purchased from Santa Cruz Biotechnology, Inc (Dallas, TX, USA). Primary antibodies anti‐acetylated H3 at lysine 9 (C5B11) (H3K9ac, #9649, 1:1000), anti‐histone H3 (D1H2) (#4499T, 1:1000) as well as HRP‐linked secondary anti‐rabbit IgG antibody (7074S) were purchased from Cell Signaling Technology (Danvers, MA, USA). HRP‐linked secondary anti‐mouse IgG (Fc specific) antibody (A0168) was purchased from Merck KGaA (Darmstadt, Germany). Loading control antibody, anti‐ß‐actin (AC‐15) (A5441, 1:2000), was obtained from Sigma‐Aldrich (St. Louis, MO, USA).

### FACS of Bodipy 495/503‐Stained Cells

0.5 × 10^5^ HCT116 and HCTR or Capan‐1 and CapanR cells were seeded in 12‐well plates in 500 µL cell culture medium and allowed to adhere overnight. Cells were either left untreated or treated with BOLD‐100, the corresponding amount of DMSO, triacsin C, or OA. LDs were stained with 0.5 µM of Bodipy 495/503^[^ ^13^, ^71^ ^]^ (D3922, Thermo Fisher Scientific, Waltham, MA, USA) for 15 min in the dark under 5% CO_2_ atmosphere at 37 °C. Cells were washed with ice‐cold PBS, trypsinized, washed again, re‐suspended in FACS‐PBS (7.81 mM Na_2_HPO_4_ × 2H_2_O, 1.47 mM KH_2_PO_4_, 2.68 mM KCl and 0.137 M NaCl) and transferred into FACS‐tubes. Bodipy 495/503 fluorescence intensity was measured on an LSRFortessa flow cytometer (BD Biosciences, East Rutherford, NJ, USA) with the FITC (b530/30 nm) bandpass emission filter. Flowing Software (University of Turku, Finland) was used for data analysis. Data are depicted as fluorescence intensities relative to respective controls.

### Time‐Lapse Microscopy (Live‐Cell Microscopy)

HCT116 and HCTR cells were seeded at a cell number of 1 × 10^5^ in 8‐well ibiTreat 1.5 polymer µ‐slides (80 826, ibidi GmbH, Gräfelfing, Germany) in 300 µL medium and incubated overnight. LDs were stained with 0.5 µM Bodipy 495/50 for 15 min and filming was initiated immediately after parameter setting. Live‐cell images were taken every 15 min with a PCO Edge 4.2 sCMOS camera using the imaging software VisiView® on the Visitron Systems 10 live‐cell microscope (Puchheim, Germany) at 40x magnification in a humidified incubation chamber ensuring stable cell culture conditions throughout the experiment. Lumencor (Beaverton, USA) spectra color LEDs were used for fluorescence illumination (475/34 nm excitation and 525/50 nm emission filter). Representative images of bright field, Bodipy 493/503‐stained, or merged channels are displayed.

### Seahorse XF Analyses

Seahorse Mito Stress Test (Seahorse XFp Cell Mito Stress Test Kit, Agilent, USA) was used for the measurement of the extracellular OCR and ECAR and was performed according to manufacturer's recommendations. Cells were seeded into 96‐well plates (XFe96/XF Pro Cell Culture Microplates, Agilent, USA) at a cell density of 2 × 10^4^ cells well^−1^ in 80 µL of cell culture medium supplemented with 10% FBS and cultured overnight. 3 h prior to measurement, cells were treated with 100 µM of BOLD‐100 or solvent control. After the incubation period, the medium was replaced by Seahorse XF DMEM assay medium (pH 7.4, Agilent, USA) supplemented with 10 mM glucose, 2 mM glutamine, and 1 mM pyruvate and incubated for another hour in a CO_2_‐free incubator at 37 °C. The contents of the Seahorse Mito Stress Test vials were reconstituted with Seahorse XF DMEM assay medium before use. The Seahorse Mito Stress Test reagents were sequentially added from the injection ports of the sensor cartridges (XFe96/XF Pro sensor cartridges, Agilent, USA) to a final concentration of: oligomycin 1.5 µM, FCCP 1 µM, and R/A 0.5 µM plus 4 µM Hoechst 33258 (1 mg mL^−1^ in PBS, for quantification of cell numbers). Following Seahorse analyses, for normalization cells were imaged and Hoechst fluorescence was measured in the DAPI channel using the Cytation 5 Cell Imaging Multimode Reader (BioTek as part of Agilent, USA). Data were processed with the Seahorse Wave Pro Software (10.0.1, Agilent, USA). OCR and ECAR levels are displayed per 1.000 cells.

### In vivo Evaluation of Xenograft Growth and Therapy Response

12‐week‐old male SCID mice were purchased from Envigo Laboratories (San Pietro al Natisone, Italy). The animals were kept in a controlled environment under pathogen‐free conditions and 12 h‐alternating light cycles, according to the FELASA guidelines. 1 × 10^6^ HCT116 or HCTR cells in 50 µL of serum‐deprived RPMI‐1640 medium were injected subcutaneously (s.c.) into the right flank of the animals. Tumor growth was assessed regularly by caliper measurement and well‐being of the animals was followed over‐time. Body weight was recorded regularly. Tumor volumes were calculated by (length×width^2^)/2. Mice received BOLD‐100 treatment 30 mg kg^−1^ i.v. on days 12, 15, 18, 21 after injection with tumor cells. BOLD‐100 was dissolved in 0.9% NaCl solution and was mixed with mouse serum immediately prior i.v. application to avoid local skin reaction. Sodium salt of etomoxir was dissolved in 0.9% NaCl solution prior to intra peritoneal (i.p.) injection. Mice were treated with 30 mg kg^−1^ of etomoxir on days 12, 13, 14, 15, as well as 18, 19, 20, 21. Solvent control groups were treated with a mixture of 0.9% NaCl and mouse serum in a ratio 1:1 i.v., corresponding to the treatment scheme of BOLD‐100, and with 0.9% NaCl solution i.p. equivalent to the therapy with etomoxir. For in vivo therapy combination, orlistat was dissolved consecutively in 10% DMSO, 40% polyethylene glycol (PEG)‐400, 5% Tween 80 (both Sigma–Aldrich, St. Louis, MO, USA), and 45% of 0.9% NaCl solution prior to i.p. injection. Mice were treated with 240 mg kg^−1^ of orlistat on days 11, 12, 13, 14, as well as 17, 18, 19, 20. BOLD‐100 treatment was applied as described above on days 12, 14, 18, 20. Solvent control groups were treated with a mixture of 0.9% NaCl and mouse serum in a ratio 1:1 i.v. corresponding to the treatment scheme of BOLD‐100, and with 10% DMSO, 40% PEG−400, 5% Tween (both Sigma–Aldrich, St. Louis, MO, USA), and 45% of 0.9% NaCl solution i.p. equivalent to the therapy with orlistat. Animals were sacrificed upon signs of reduced well‐being or ulceration or when the tumor exceeded a length > 20 mm in one dimension. All animal experiments were controlled by the Ethics Committee for the Care and Use of Laboratory Animals at the Medical University Vienna (proposal numbers: BMBWF‐66.009/0157‐V/3b/2019).

### Histological Evaluation

Tumors and organs were formalin‐fixed in 4% formaldehyde for 24 h (Carl Roth, # P087.3) and paraffin‐embedded using a KOS machine (Milestone Medical, Sorisole, Italy). For histological evaluation, embedded tumors and organs were cut into 4 µm thick sections and used for H&E staining. Tissue was deparaffinized, rehydrated, and stained with H&E by routine procedures.

### Immunohistochemistry

Paraffin‐embedded tissue was cut into 4 µM thick sections. IHC was performed as described previously using anti‐H3K9ac antibody (1:800 dilution; #9649, Cell Signaling Technology, Danvers, MA, USA). Binding of primary antibodies was detected with the UltraVision LP detection system according to the manufacturer's instructions (Thermo Fisher Scientific, Waltham, MA, USA), followed by incubation with 3,3′‐diaminobenzidine (cat.no. K3468, Dako GmbH, Jena, Germany) and counterstaining with hematoxylin Gill III (cat.no. 1.05174.0500, Merck KGaA, Darmstadt, Germany).

### Lactate Accumulation/Secretion Measurements

HCT116 and HCTR cells were seeded in triplicates at a density of 5 × 10^5^ cells well^−1^ in 6 well plates in 2 mL of medium and incubated overnight. Medium was replaced and cells were treated with 100 µM of BOLD‐100 or DMSO for 24 h. Lactate secretion was measured in the supernatant using the ARKRAY LACTATE PRO 2 LT‐1730 test meter (SPORT BUCK GmbH, Kempten, Germany). For intracellular lactate accumulation, cells were collected, washed with PBS, lysed in 20 µL of bidistilled H_2_O by repeated freeze and thaw cycles, and sonicated for lactate release. 2.5 µL of the solution were measured with the lactate test meter. Intracellular lactate levels were normalized to protein concentration.

### ESI and MS

The reactions between BOLD‐100 or etomoxir (as positive control) with CoA were assessed by diluting stock solutions to 50 µM in ammonium carbonate (pH = 7.4, 4 mM) at equimolar ratios. Reaction aliquots were taken after 30 min, 4 and 24 h of incubation at 37 °C and under constant shaking. Aliquots were immediately snap frozen until analysis. MS data was acquired on an Amazon Speed ETD (Bruker Daltonics, Bremen, Germany) by direct infusion of analytes diluted to 1–5 µM with aqueous solution (LC‐MS grade water, Fluka). The instrument parameters were as follows: 4.5 kV capillary voltage, 500 V end plate offset, 3 bar nebulizer, 5 L min^−1^ dry gas, 180 °C dry temperature, 30 000 ICC target and m/z 100–1400 scan range. Mass spectra were recorded over 0.4 min and averaged in positive and negative ion modes.

### NMR

^1^H NMR spectra (500.32 MHz) were recorded on an Avance NEO 500 NMR spectrometer (Bruker BioSpin, Bremen, Germany) with BOLD‐100 and CoA at concentrations of 2.5 mM in D_2_O containing 0.5% DMSO‐d6. NMR spectra were evaluated using TopSpin Version 3.6.3. (Bruker BioSpin, Bremen, Germany). The reaction between BOLD‐100 and CoA was followed every 30 min during the first 4 h and then every hour up to 16 h. The reaction mixture turned from clear orange to yellow‐green opaque. The intensities of the NMR signals between CoA and BOLD‐100 confirmed a 1:1 molar ratio.

### Metabolomics

Metabolomics were performed as described before.^[^ ^20^, ^21^ ^]^ In brief, HCT116 and HCTR cells were cultured at a density of 0.25 × 10^6^ cells mL^−1^ in 1 mL MC10 medium in 12‐well plates and left to recover overnight at 37 °C and 5% CO_2_. Via medium exchange, cells were treated with 100 µM of BOLD‐100 or DMSO for 24 h. Cells were washed 3 times with PBS, quenched with liquid nitrogen, and stored on −80 °C until further use. Metabolomic analyses were performed as described before.^[^ ^20^ ^]^ In short, liquid chromatography high resolution mass spectrometry (LC‐MS) measurement with Thermo Scientific Q Exactive HF quadrupole‐Orbitrap mass spectrometer was utilized in full mass scan mode (both positive and negative ionization‐mode) and a resolution of 120 000. External calibration with fully labelled ^13^C internal standards ISOtopic solutions (Vienna, Austria) was used for quantification. Obtained absolute metabolite amounts (pmol) were normalized to total protein content of the respective well (µg) with the Micro BCA™ Protein Assay Kit (Thermo Fisher Scientific, Waltham, MA, USA), according to manufacturer's recommendations.

### Statistical Analyses

Data were analysed using GraphPad Prism software (version 8.0.1, La Jolla, CA, 381 USA). If not stated otherwise in the figure legends, one out of at least three independent experiments in triplicates was displayed. Each data point represents the mean ± standard deviation (SD) of triplicate values. Statistical evaluation of significance was performed using unpaired student's t‐test as well as one‐ or two‐way analysis of variance (ANOVA) with Bonferroni's or Tukey's multiple comparisons post‐tests. Tumor volumes are given as mean ± SEM and were tested for statistical significance using two‐way ANOVA with Sidak's or Tukey's multiple comparisons test. *p*‐values below 0.05 were considered statistically significant: *p<0.05, **p<0.005, ***p<0.0005, ****p<0.0001, ns: non‐significant.

### Ethics Approval

All animal experiments were controlled by the Ethics Committee for the Care and Use of Laboratory Animals at the Medical University Vienna (proposal numbers: BMBWF‐66.009/0157‐V/3b/2019).

## Conflict of Interest

The authors declare no conflict of interest.

## Author Contributions

D.B. and W.B. designed the study and conceptualized the manuscript. D.B., T.M., B.S.‐A., C.P., B.R., M.R., M.S., N.S., N.J.‐M.R., and S.M.M.‐M. conducted the experiments in this study. D.B., C.P., T.Mo., W.M.S., and M.R. performed the data analysis. D.B. and W.B. wrote the manuscript. S.M.M.‐M., K.N., G.K., N.S., N.J.‐M.R., W.M.S., and B.K.K. participated in discussions. T.M., B.S.‐A., C.P., T.Mo., M.R., M.S., N.S., N.J.‐M.R., S.M.M.‐M., P.H., B.K.K., and W.B. reviewed the manuscript. All authors read and approved the final manuscript.

## Supporting information

Supporting Information

## Data Availability

The data that support the findings of this study are available from the corresponding author upon reasonable request.
