# Supplementary material for: The Lipid Metabolism as Target and Modulator of BOLD‐100 Anticancer Activity: Crosstalk with Histone Acetylation
Source: Adv Sci (Weinh). 2023 Sep 26;10(32):2301939. doi: 10.1002/advs.202301939 (PMC10646284; doi:10.1002/advs.202301939)
Supplement: Supplementary file 1 — Supporting Information [file ADVS-10-2301939-s001.pdf]

ADVANCED SCIENCE
Open Access

Supporting Information

for *Adv. Sci.*, DOI 10.1002/advs.202301939

The Lipid Metabolism as Target and Modulator of BOLD-100 Anticancer Activity: Crosstalk with Histone Acetylation

*Dina Baier, Theresa Mendrina, Beatrix Schoenhacker-Alte, Christine Pirker, Thomas Mohr, Mate Rusz, Benedict Regner, Martin Schaier, Nicolas Sgarioto, Noël J.-M. Raynal, Karin Nowikovsky, Wolfgang M. Schmidt, Petra Heffeter, Samuel M. Meier-Menches, Gunda Koellensperger, Bernhard K. Keppler and Walter Berger**

Supporting Information

The lipid metabolism as target and modulator of BOLD-100 anticancer activity: crosstalk with histone acetylation.

Dina Baier[1,2,3], Theresa Mendrina[1,2,3], Beatrix Schoenhacker-Alte[1,2,3], Christine Pirker[1,3], Thomas Mohr[1,5,9], Mate Rusz[2,3,4], Benedict Regner[6], Martin Schaier[4], Nicolas Sgarioto[8], Noël J.-M. Raynal[8], Karin Nowikovsky[6,7], Wolfgang M. Schmidt[10], Petra Heffeter[1,3], Samuel M. Meier-Menches[2,4,5], Gunda Koellensperger[4], Bernhard K. Keppler[2,3], Walter Berger[1,3]*

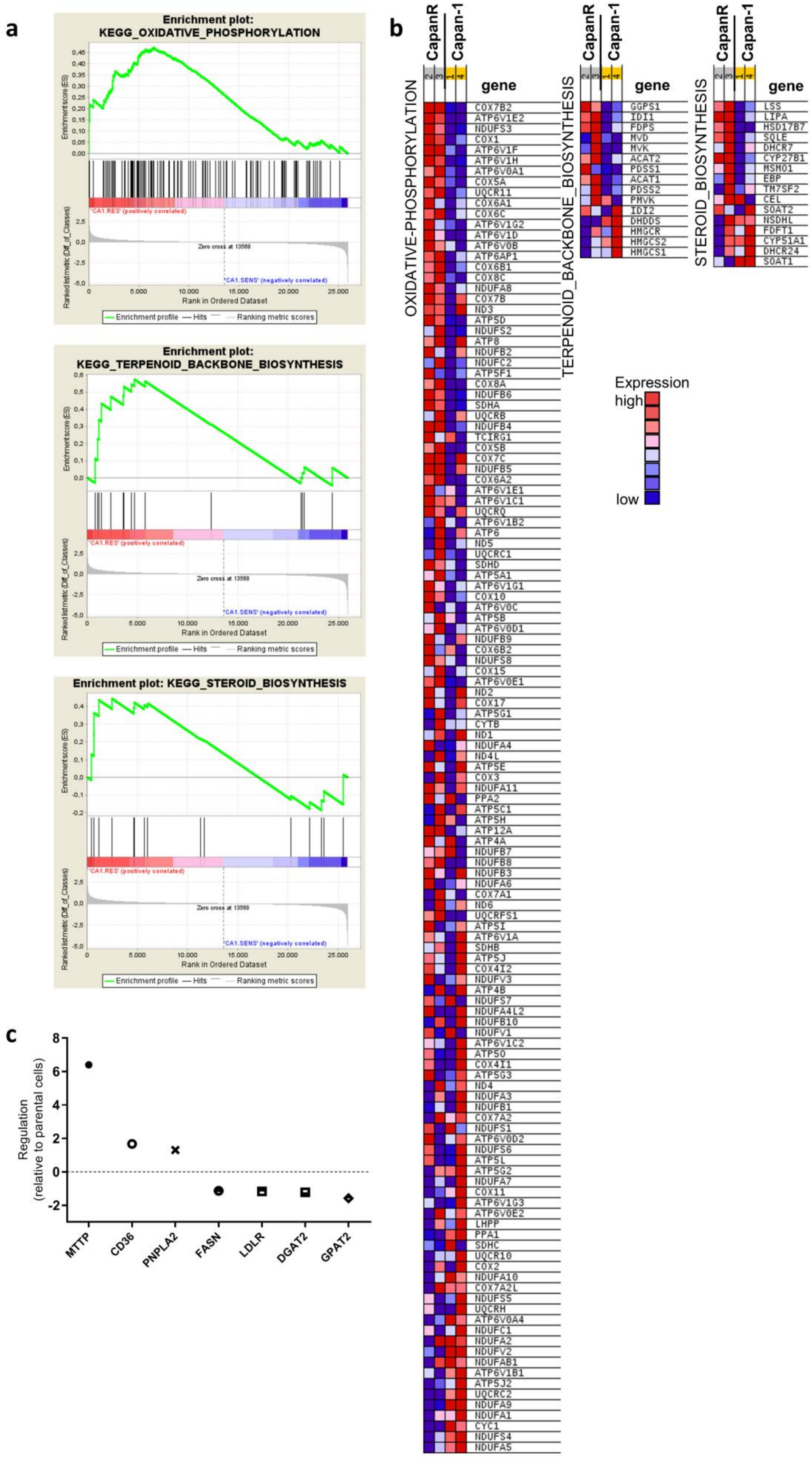
a
Enrichment plot: KEGG_OXIDATIVE_PHOSPHORYLATION
Enrichment plot: KEGG_TERPENOID_BACKBONE_BIOSYNTHESIS
Enrichment plot: KEGG_STEROID_BIOSYNTHESIS
Enrichment score (ES)
'CA1.RES' (positively correlated)
'CA1.SENS' (negatively correlated)
Zero cross at 13568
Ranked list metric (Diff_of_Classes)
Rank in Ordered Dataset
Enrichment profile
Hits
Ranking metric scores
b
CapanR
Capan-1
gene
OXIDATIVE-PHOSPHORYLATION
COX7B2 ATP6V1E2 NDUFS3 COX1 ATP6V1F ATP6V1H ATP6V0A1 COX5A UQCR11 COX6A1 COX6C ATP6V1G2 ATP6V1D ATP6V0B ATP6AP1 COX6B1 COX8C NDUFA8 COX7B ND3 ATP5D NDUFS2 ATP8 NDUFB2 NDUFC2 ATP5F1 COX8A NDUFB6 SDHA UQCRB NDUFB4 TCIRG1 COX5B COX7C NDUFB5 COX6A2 ATP6V1E1 ATP6V1C1 UQCRQ ATP6V1B2 ATP6 ND5 UQCRC1 SDHD ATP5A1 ATP6V1G1 COX10 ATP6V0C ATP5B ATP6V0D1 NDUFB9 COX6B2 NDUFS8 COX15 ATP6V0E1 ND2 COX17 ATP5G1 CYTB ND1 NDUFA4 ND4L ATP5E COX3 NDUFA11 PPA2 ATP5C1 ATP5H ATP12A ATP4A NDUFB7 NDUFB8 NDUFB3 NDUFA6 COX7A1 ND6 UQCRFS1 ATP5I ATP6V1A SDHB ATP5J COX4I2 NDUFV3 ATP4B NDUFS7 NDUFA4L2 NDUFB10 NDUFV1 ATP6V1C2 ATP5O COX4I1 ATP5G3 ND4 NDUFA3 NDUFB1 COX7A2 NDUFS1 ATP6V0D2 NDUFS6 ATP5L ATP5G2 NDUFA7 COX11 ATP6V1G3 ATP6V0E2 LHPP PPA1 SDHC UQCR10 COX2 NDUFA10 COX7A2L NDUFS5 UQCRH ATP6V0A4 NDUFC1 NDUFA2 NDUFV2 NDUFAB1 ATP6V1B1 ATP5J2 UQCRC2 NDUFA9 NDUFA1 CYC1 NDUFS4 NDUFA5
TERPENOID_BACKBONE_BIOSYNTHESIS
GGPS1 IDI1 FDPS MVD MVK ACAT2 PDSS1 ACAT1 PDSS2 PMVK IDI2 DHDDS HMGCR HMGCS2 HMGCS1
STEROID_BIOSYNTHESIS
LSS LIPA HSD17B7 SQLE DHCR7 CYP27B1 MSMO1 EBP TM7SF2 CEL SOAT2 NSDHL FDFT1 CYP51A1 DHCR24 SOAT1
Expression
high
low
c
Regulation (relative to parental cells)
MTTP
CD36
PNPLA2
FASN
LDLR
DGAT2
GPAT2

Figure S1. Acquired BOLD-100 resistance of Capan-1 cells is associated with transcriptional deregulation of the lipid metabolism. a) GSEA identifies “OXIDATIVE_ PHOSPHORYLATION” (nominal *p*-value$<E^{-7}$; FDR q-value=0.121) and “TERPENOID_BACKBONE_BIOSYNTHESIS” (nominal *p*-value=0.169; FDR q-value=0.652) as the third and sixth top enriched gene sets, respectively, from the KEGG database in CapanR vs. Capan-1 cells. b) Heatmaps of respective top enriched gene sets identified from GSEA in CapanR vs. Capan-1 cells. c) Regulation of specific genes involved in fatty acid and cholesterol metabolism of CapanR vs. Capan-1 cells (indicated as dashed line at 0). mRNA levels with a fold change$>|1.1|$ were considered for graphical display.

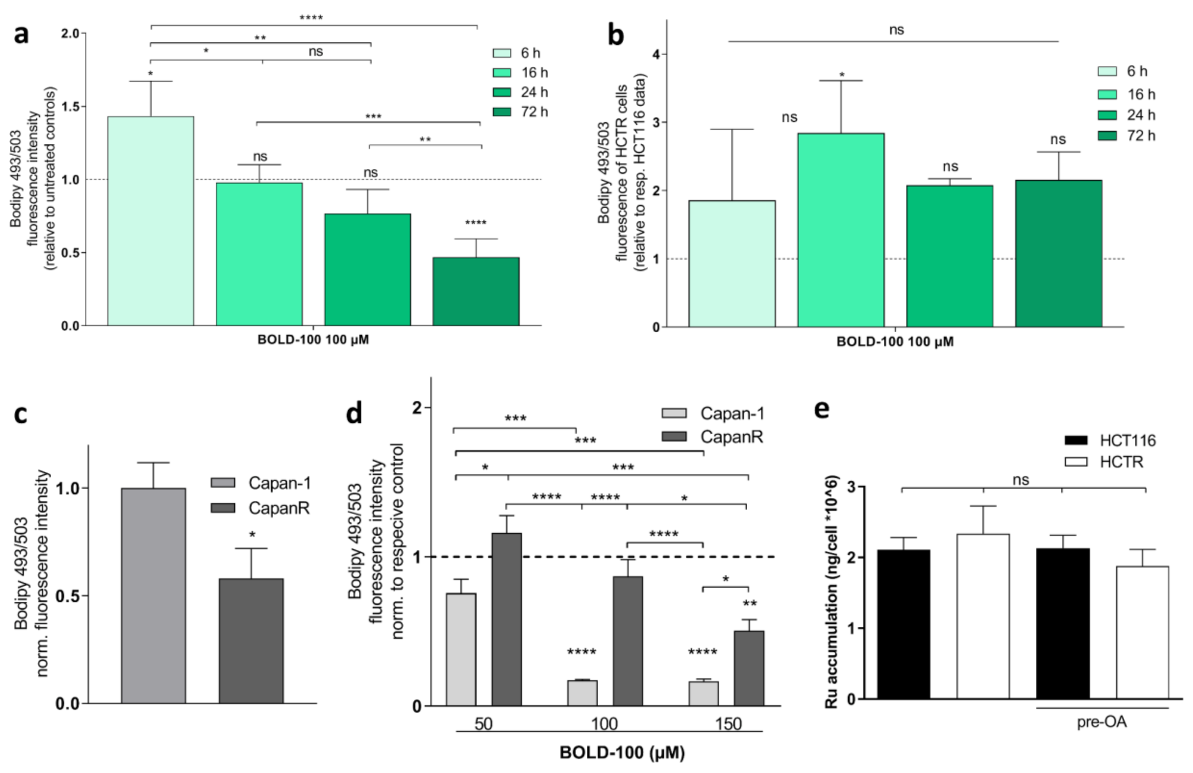

Figure S2. Cellular lipid content is modulated by and affects the anticancer activity of BOLD-100. a) Fluorescence intensity of parental HCT116 cells after treatment with 100 µM of BOLD-100 for indicated time frames normalized to controls (depicted as dashed line) determined by flow cytometry after staining with 0.5 µM Bodipy 493/503 for 15 min. One-way ANOVA with Bonferroni's multiple comparisons test: *p<0.05, ***p<0.0001, ****p<0.0001, ns: non-significant. b) Bodipy 493/503 fluorescence intensity of HCTR cells after treatment with 100 µM of BOLD-100 for indicated time frames normalized to corresponding HCT116 cells (depicted as dashed line) determined by flow cytometry after staining with 0.5 µM of Bodipy 493/503 for 15 min. One-way ANOVA with Bonferroni's multiple comparisons test: *p<0.05, ns. c) Normalized fluorescence intensity of Capan-1 and CapanR cells determined by FACS after staining with 0.5 µM of Bodipy 493/503. Unpaired two-tailed student´s t-test: *p<0.05. d) Fluorescence intensity of Capan-1 and CapanR cells treated with the indicated concentrations of BOLD-100 for 72 h determined by FACS after staining with 0.5 µM of Bodipy 493/503 for 15 min. Data are normalized to the respective

controls. Two-way ANOVA with Tukey´s multiple comparisons test: *$p<0.05$, **$p<0.005$, ***$p<0.0005$, ****$p<0.0001$. e) ICP-MS analyses of whole cell lysates of BOLD-100-sensitive and -resistant HCT116 cells after 72 h pre-treatment with 100 µM of OA and 24 h of treatment with 100 µM of BOLD-100. One-way ANOVA with Bonferroni’s multiple comparisons test: ns.

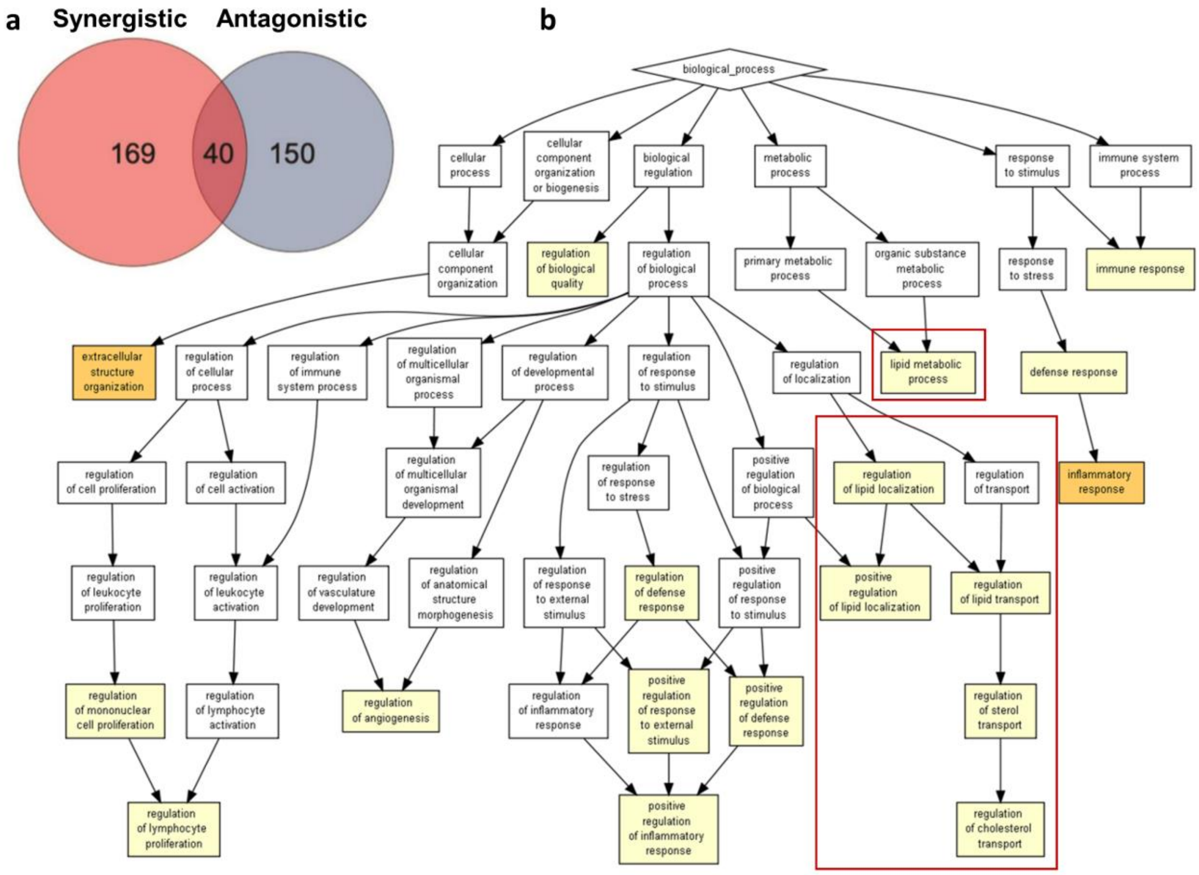

Figure S3. Drugs synergizing with BOLD-100 impact lipid metabolic processes. a) Venn diagram of gene selection process of unbiased drug-gene interaction screens of 1760 compounds where 129 genes were associated with synergistic drugs and 110 genes were associated with antagonistic drugs. b) Biological process analysis of genes associated with synergistic hits. Lipid processing- and regulation of sterol transport associated hits are displayed in red boxes.

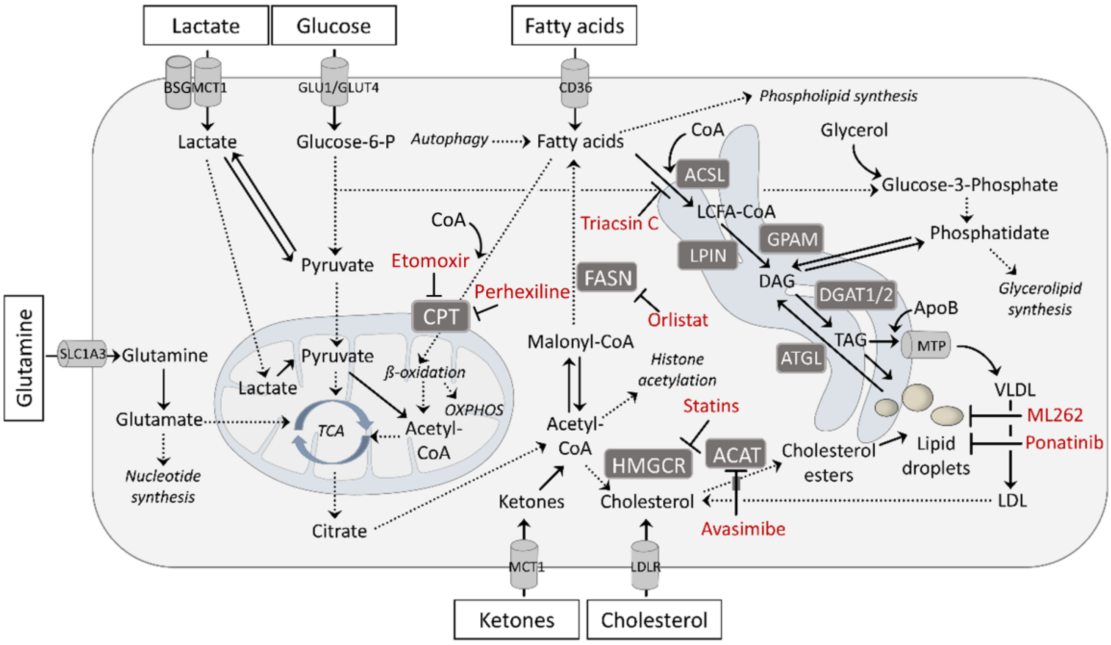

Figure S4. Scheme of drug-respective modes of action. Drugs are indicated in red. Building blocks for schematic representation were provided by © Motifolio (license holder: T. Mohr).

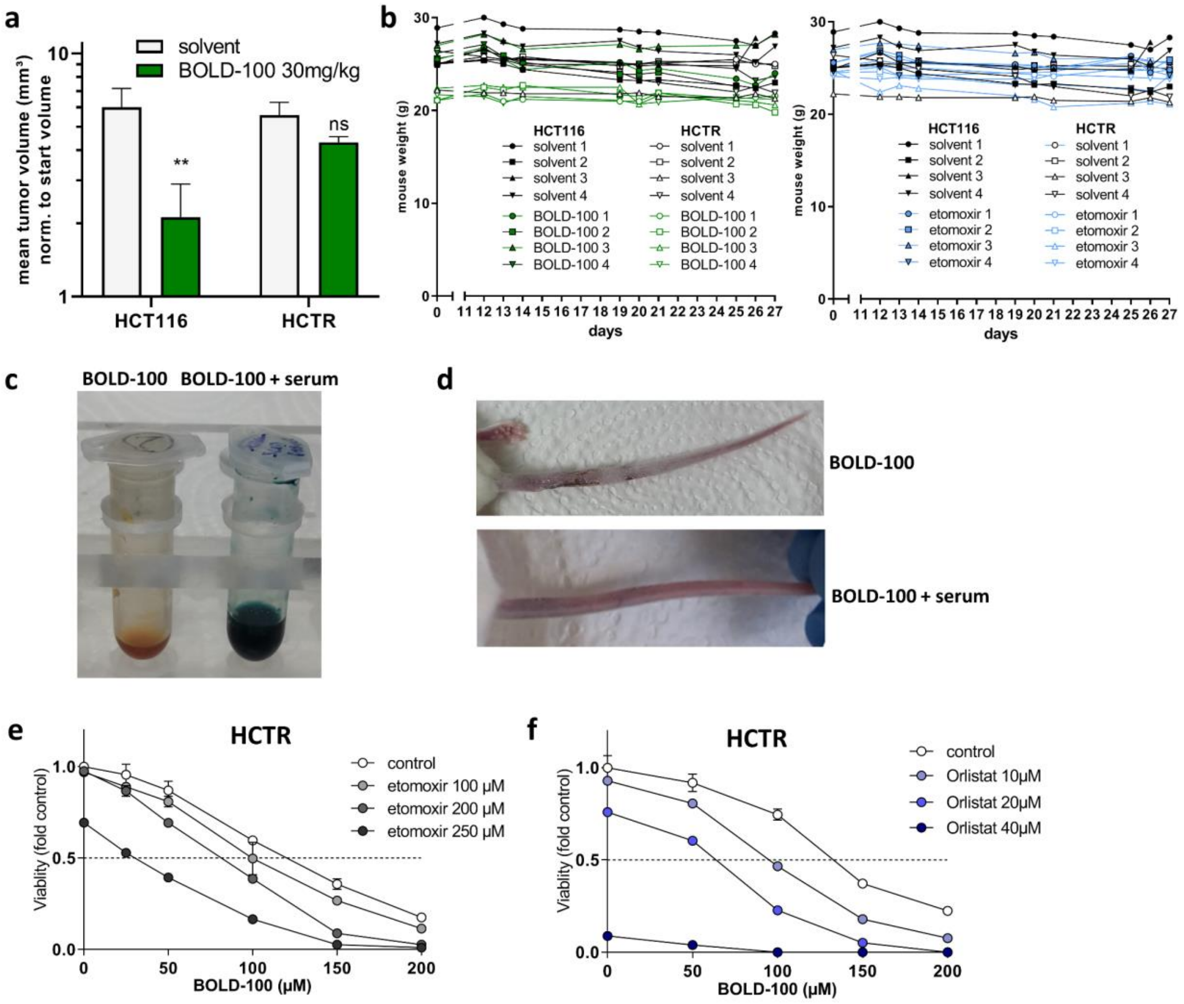

Figure S5. BOLD-100 significantly reduced HCT116, but not HCTR xenograft growth, and did not decrease mouse weight. a) Impact of BOLD-100 on HCT116 or HCTR xenograft growth depicted representatively by the tumor volume at day 27 after inoculation presented as means ± SEM. Statistical significance was tested by two-way ANOVA with Sidak's multiple comparisons test: $*p<0.05$; n=4 per experimental group. b) Monitoring of individual mouse weights over 27 days of the course of the in vivo experiment. Left: BOLD-100 compared to solvent control, right graph: etomoxir compared to solvent control. c) BOLD-100 dissolved in 0.9 % NaCl (left, yellow-brownish) as compared to BOLD-100 dissolved in 0.9 % NaCl co-incubated with mouse serum for 2 min (right, green-blueish). d) Tails of mice treated without (upper) or with (lower) serum pre-incubation before treatment. e, f) Cell viability assays after

72 h of treatment with BOLD-100 in combination with e) etomoxir or f) orlistat at indicated concentrations were performed. One representative of three independent experiments is shown.

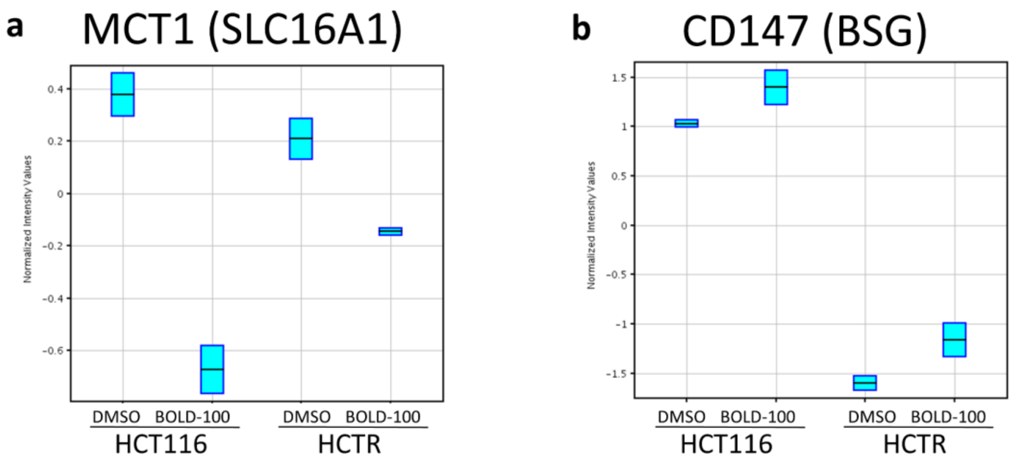

Figure S6. mRNA expression changes of MCT1 and its cofactor CD147 in response to BOLD-100. a) Relative mRNA expression levels of *SLC16A1* and b) *BSG* in HCT116 and HCTR cells after 6 h of treatment with or without 100 µM of BOLD-100.

a wt

MAAALFVLLGFALLGTHGASGAAGTVFTTVEDLGSKILLTCSLNDSATEVTGHRWLKGGVVLKEDALPGQKTEFKVDSDD
QWGEYSCVFLPEPMGTANIQLHGPPRVKAVKSSEHINEGETAMLVCKSESVPPVTDWAWYKITDSEDKALMNGSESRFF
VSSSQGRSELHIENLNMEADP**G**QYRCNGTSSKGSDQAIITLRVRSHLAALWPFLGIVAEVLVLVTIIFIYEKRRKPEDVLDDD
DAGSAPLKSSGQHQNDKGKNVRQRNSS

b mut

MAAALFVLLGFALLGTHGASGAAGTVFTTVEDLGSKILLTCSLNDSATEVTGHRWLKGGVVLKEDALPGQKTEFKVDSDD
QWGEYSCVFLPEPMGTANIQLHGPPRVKAVKSSEHINEGETAMLVCKSESVPPVTDWAWYKITDSEDKALMNGSESRFF
VSSSQGRSELHIENLNMEADP**P**ASTGATAPAPRAPTRPSSRSACAATWPPSGPSWASWLRCWCWSPSSSSTRSAGSPRT
SWMMTTPALHP*

Figure S7. WES reveals frameshift-inducing insertion mutation of *BSG* in HCTR cells. Affected CD147 protein a) reference (wt) vs. b) predicted sequence (mut) of *BSG* NM_198589.3:exon5:c.539_540dup: p.(Gly181Profs*70) in HCTR cells; blue box: TM, green box: cytoplasmic domain of reference wt protein sequence.

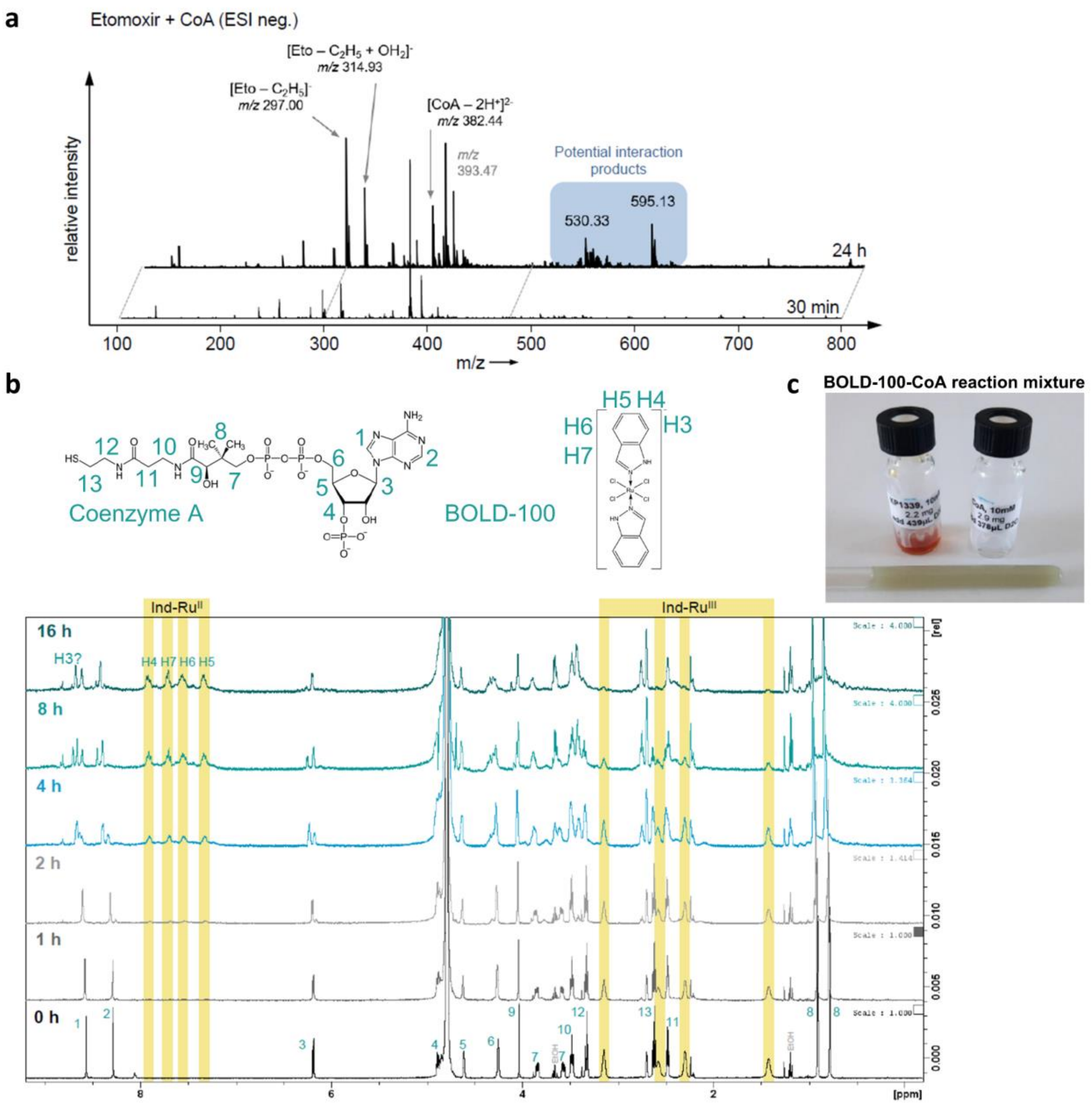

Figure S8. Co-incubation of BOLD-100 with CoA leads to spontaneous adduct formation in a similar mode as etomoxir. a) Mass spectra of the interaction between etomoxir and CoA (1:1 molar ratio) acquired in the negative ion mode after the indicated incubation times. b) 1H NMR spectra of CoA and BOLD-100 (1:1 molar ratio) acquired over the indicated incubation time. c) Characteristic color shift of the BOLD-100-CoA reaction mixture (reaction tube).

a

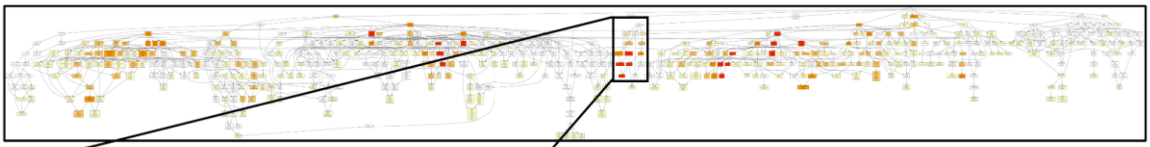

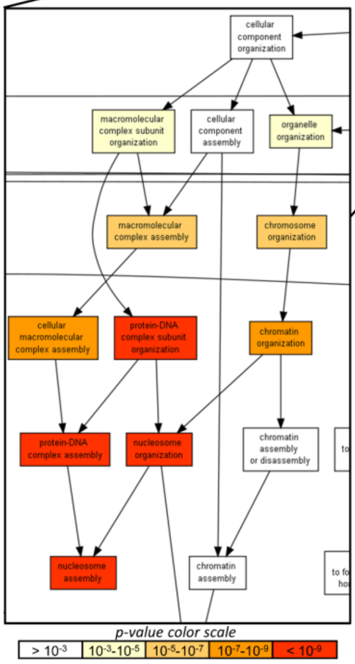

GO term	Description	p-value	FDR q-value
GO:0006334	nucleosome assembly	4.98E-22	5.87E-18
GO:0065004	protein-DNA complex assembly	1.24E-20	7.31E-17
GO:0034728	nucleosome organization	8.98E-20	3.53E-16
GO:0071824	protein-DNA complex subunit organization	1.18E-18	3.47E-15

HCT116 BOLD-100 6h vs 0h
Top 4 enriched GO-Terms

Genes
HIST1H1E - histone cluster 1, h1e
HIST2H2AC - histone cluster 2, h2ac
HIST1H1D - histone cluster 1, h1d
HIST1H1C - histone cluster 1, h1c
HIST2H2AA4 - histone cluster 2, h2aa4
H1F0 - h1 histone family, member 0
HIST1H2AB - histone cluster 1, h2ab
H1FX - h1 histone family, member x
HIST1H2BH - histone cluster 1, h2bh
HIST1H2BC - histone cluster 1, h2bc
HIST1H2BL - histone cluster 1, h2bl
HIST1H2BM - histone cluster 1, h2bm
HIST2H2AB - histone cluster 2, h2ab
HIST1H2AH - histone cluster 1, h2ah
HIST2H2BF - histone cluster 2, h2bf
HIST1H2BK - histone cluster 1, h2bk
HIST2H4B - histone cluster 2, h4b
HIST1H3F - histone cluster 1, h3f
HIST1H2BO - histone cluster 1, h2bo
HIST1H2BD - histone cluster 1, h2bd
HIST1H2AK - histone cluster 1, h2ak
HIST1H2BB - histone cluster 1, h2bb
HIST1H2AD - histone cluster 1, h2ad

b

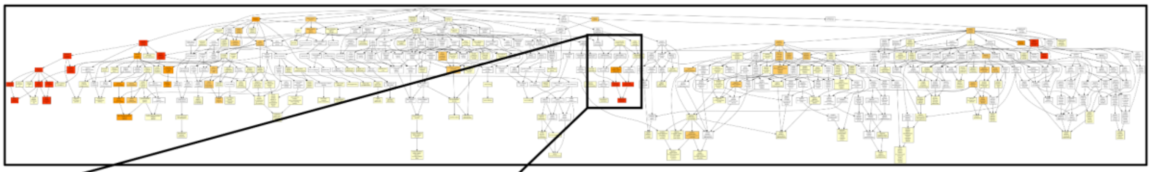

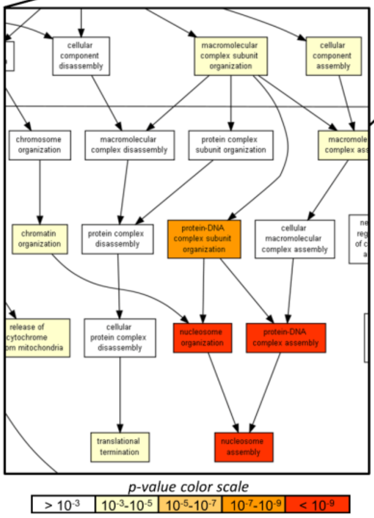

GO term	Description	P-value	FDR q-value
GO:0006334	nucleosome assembly	9.72E-12	9.54E-9
GO:0065004	protein-DNA complex assembly	1.26E-10	1.06E-7
GO:0034728	nucleosome organization	5.96E-10	4.68E-7
GO:0071824	protein-DNA complex subunit organization	1.27E-9	9.36E-7

Capan-1 BOLD-100 6h vs 0h
Top 12, 14, 15, 16 enriched GO-Terms

Figure S9. BOLD-100 treatment induces enrichment of GO-terms associated with histone regulation and organization. Enrichment of selected GO-terms after 6 h of treatment with BOLD-100 compared to untreated a) HCT116 or b) Capan-1 cells.

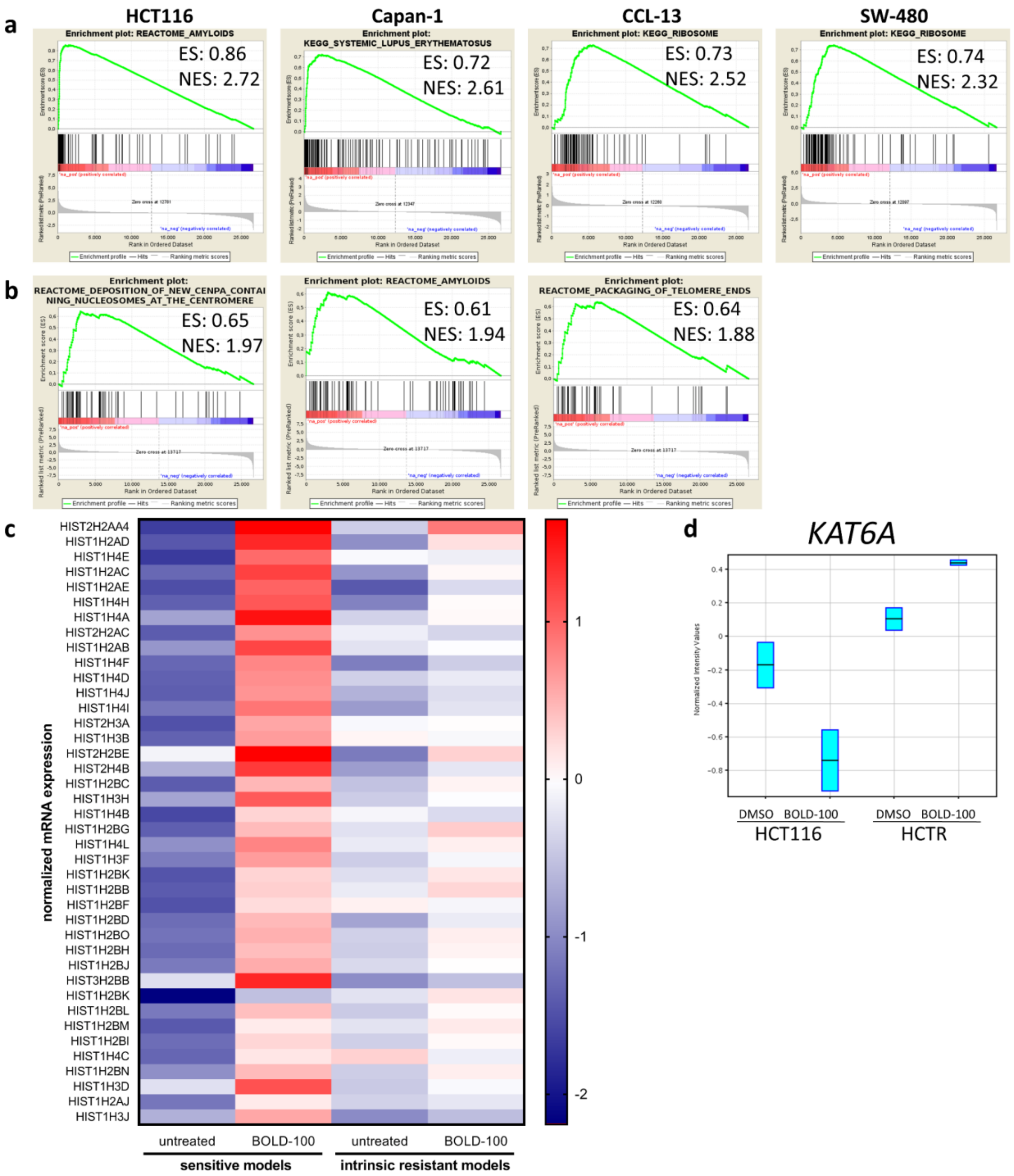

Figure S10. BOLD-100 treatment upregulates histone gene expression of sensitive but not intrinsically resistant cell models. a) GSEA identifies "REACTOME_AMYLOIDS" and "SYSTEMIC_LUPUS_ERYTHEMATOSUS" as top enriched gene sets under 6h BOLD-100 treatment compared to untreated controls in sensitive HCT116 and Capan-1 cells, respectively. In intrinsically resistant CCL-13 and SW480 cells, GSEA identifies "KEGG_RIBOSOME" as

top enriched gene set under BOLD-100 treatment. b) GSEA identifies histone-associated gene sets as top three enriched in untreated pooled intrinsically resistant as compared to pooled sensitive models. c) Heat map displaying normalized mRNA expression of histone genes of the top enriched gene set "REACTOME_AMYLOIDS" in pooled sensitive vs. intrinsically resistant models in response to BOLD-100 vs. untreated control (FC>2). d) Relative mRNA expression levels of *KAT6A* in HCT116 and HCTR cells after 6 h of treatment with or without 100 µM of BOLD-100.

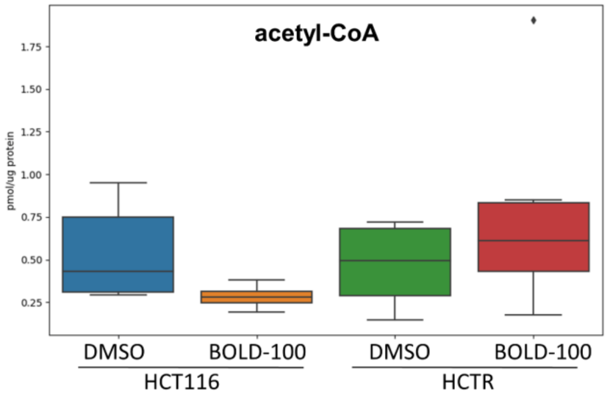

Figure S11. BOLD-100 treatment reduces acetyl-CoA only in parental HCT116 cells. Impact of BOLD-100 (24h, 100 µM) or solvent control DMSO exposure on the level on acetyl-CoA (pmol/µg protein, n=6 biological replicates) in HCT116 and HCTR cells as determined by metabolomics analysis.
